# Supplementary material for: Patterns of emergency admissions for ambulatory care sensitive conditions: a spatial cross-sectional analysis of observational data
Source: BMJ Open. 2020 Nov 4;10(11):e039910. doi: 10.1136/bmjopen-2020-039910 (PMC7643517; doi:10.1136/bmjopen-2020-039910)
Supplement: Supplementary data [file bmjopen-2020-039910supp001.pdf]

Supplementary Tables

Table A1. Data sources
Table A2. ICD10 codes defining ACSCs
Table A3. Number and annual growth rate of ACSC emergency admissions 2004-2017
Table A4. Average spatial correlation of ISARs 2004-2017.
Table A5. Clustering of ISARs 2004-2017
Table A6. Transition probabilities between clusters 2004 to 2017.

Table A1. Data sources

Data	Data source
Number of patients by age and gender	NHS Digital<br>http://content.digital.nhs.uk/workforce
2015 Index of Multiple Deprivation from Neighbourhood Statistics	Office for National Statistics<br>http://www.neighbourhood.statistics.gov.uk/dissemination/
Attribution Data Set	NHS Digital<br>http://content.digital.nhs.uk/
2017 CCG boundaries	https://data.gov.uk/

Santos R, *et al. BMJ Open* 2020; 10:e039910. doi: 10.1136/bmjopen-2020-039910

Table A2. ICD10 codes for ACSCs and for incentivised ACSCs.

Disease group	ICD10 code	ICD 10 Name	NHS Outcomes Framework 2014/2015	Harrison et al 2013
Angina	I20	Angina pectoris	Chronic	Incentivized
Angina	I24.0	Coronary thrombosis not resulting in myocardial infarction	Acute	Incentivized
Angina	I24.8	Other forms of acute ischaemic heart disease	Acute	Incentivized
Angina	I24.9	Acute ischaemic heart disease, unspecified	Acute	Incentivized
Asthma	J45	Asthma	Chronic	Incentivized
Asthma	J46	Status asthmaticus	Chronic	Incentivized
Cardiovascular diseases	I13.0	Hypertensive heart and renal disease with (congestive) heart failure	Chronic	Incentivized
Cardiovascular diseases	I25	Chronic ischaemic heart disease	Chronic	Incentivized
Cardiovascular diseases	I48X	Atrial fibrillation and flutter	Chronic	
Cellulitis	L01	Impetigo	Acute	
Cellulitis	L02	Cutaneous abscess, furuncle and carbuncle	Acute	
Cellulitis	L03	Cellulitis	Acute	Non-incentivized
Cellulitis	L04	Acute lymphadenitis	Acute	Non-incentivized
Cellulitis	L08.0	Pyoderma	Acute	Non-incentivized
Cellulitis	L08.8	Other specified local infections of skin and subcutaneous tissue	Acute	Non-incentivized
Cellulitis	L08.9	Local infection of skin and subcutaneous tissue, unspecified	Acute	Non-incentivized
Cellulitis	L88	Pyoderma gangrenosum	Acute	Non-incentivized
Cellulitis	L98.0	Pyogenic granuloma	Acute	Non-incentivized
Cellulitis	I89.1	Lymphangitis	Acute	
Chronic obstructive pulmonary disease	J20	Acute bronchitis	Chronic	Incentivized
Chronic obstructive pulmonary disease	J41	Simple and mucopurulent chronic bronchitis	Chronic	Incentivized
Chronic obstructive pulmonary disease	J42	Unspecified chronic bronchitis	Chronic	Incentivized
Chronic obstructive pulmonary disease	J43	Emphysema	Chronic	Incentivized
Chronic obstructive pulmonary disease	J44	Other chronic obstructive pulmonary disease	Chronic	Incentivized
Chronic obstructive pulmonary disease	J47	Bronchiectasis	Chronic	Incentivized
Congestive heart failure	I11.0	Hypertensive heart disease with (congestive) heart failure	Chronic	Incentivized
Congestive heart failure	I50	Heart failure		Incentivized
Congestive heart failure	J81	Pulmonary oedema	Chronic	Incentivized
Convulsions and epilepsy	G40	Epilepsy	Chronic	Incentivized
Convulsions and epilepsy	G41	Status epilepticus	Chronic	Incentivized
Dehydration and gastroenteritis	E86	Volume depletion	Acute	Non-incentivized
Dehydration and gastroenteritis	K52.2	Allergic and dietetic gastro-enteritis and colitis		Non-incentivized

Santos R, *et al. BMJ Open* 2020; 10:e039910. doi: 10.1136/bmjopen-2020-039910

**Disease group**	**ICD10 code**	**ICD 10 Name**	**NHS Outcomes Framework 2014/2015**	**Harrison et al 2013**
Dehydration and gastroenteritis	K52.8	Other specified non-infective gastro-enteritis and colitis		Non-incentivized
Dehydration and gastroenteritis	K52.9	Non-infective gastro-enteritis and colitis, unspecified		Non-incentivized
Diabetes (hypoglycaemic)	E16.2	Hypoglycaemia, unspecified		Incentivized
Diabetes complications	E10.0–E10.8	Insulin-dependent diabetes mellitus	Chronic	Incentivized
Diseases of the blood	D51	Vitamin B12 deficiency anaemia	Chronic	
Diseases of the blood	D52	Folate deficiency anaemia	Chronic	
Ear, nose and throat infections	H66	Suppurative and unspecified otitis media	Acute	Non-incentivized
Ear, nose and throat infections	H67	Otitis media in diseases classified elsewhere	Acute	Non-incentivized
Ear, nose and throat infections	J02	Acute pharyngitis	Acute	Non-incentivized
Ear, nose and throat infections	J03	Acute tonsillitis	Acute	Non-incentivized
Ear, nose and throat infections	J04	Acute laryngitis and tracheitis	Acute	Non-incentivized
Ear, nose and throat infections	J06	Acute upper respiratory infections of multiple and unspecified sites	Acute	Non-incentivized
Ear, nose and throat infections	J31.2	Chronic pharyngitis	Acute	Non-incentivized
Gangrene	R02	Gangrene, not elsewhere classified		Non-incentivized
Hypertension	I10	Essential (primary) hypertension	Chronic	Incentivized
Hypertension	I11.9	Hypertensive heart disease without (congestive) heart failure	Chronic	Incentivized
Influenza and pneumonia	J10	Influenza due to identified influenza virus	Acute	
Influenza and pneumonia	J11	Influenza, virus not identified	Acute	
Influenza and pneumonia	J13X	Pneumonia due to Streptococcus pneumoniae	Acute	
Influenza and pneumonia	J14	Pneumonia due to Haemophilus influenzae	Acute	
Influenza and pneumonia	J15.3	Pneumonia due to streptococcus, group B	Acute	
Influenza and pneumonia	J15.4	Pneumonia due to other streptococci	Acute	
Influenza and pneumonia	J15.7	Pneumonia due to Mycoplasma pneumoniae	Acute	
Influenza and pneumonia	J15.9	Bacterial pneumonia, unspecified	Acute	
Influenza and pneumonia	J16.8	Pneumonia due to other specified infectious organisms	Acute	
Influenza and pneumonia	J18.1	Lobar pneumonia, unspecified	Acute	
Influenza and pneumonia	J18.8	Other pneumonia, organism unspecified	Acute	
Iron deficiency anaemia	D50.1	Sideropenic dysphagia	Chronic	Non-incentivized
Iron deficiency anaemia	D50.8	Other iron deficiency anaemias	Chronic	Non-incentivized
Iron deficiency anaemia	D50.9	Iron deficiency anaemia, unspecified	Chronic	Non-incentivized
Mental and behavioural disorders	F00	Dementia in Alzheimer's disease	Chronic	
Mental and behavioural disorders	F01	Vascular dementia	Chronic	
Mental and behavioural disorders	F02	Dementia in other diseases classified elsewhere	Chronic	
Mental and behavioural disorders	F03	Unspecified dementia	Chronic	

Disease group	ICD10 code	ICD 10 Name	NHS Outcomes Framework 2014/2015	Harrison et al 2013
Mental and behavioural disorders	G30.0	Alzheimer's disease with early onset	Chronic	
Mental and behavioural disorders	G30.1	Alzheimer's disease with late onset	Chronic	
Mental and behavioural disorders	G30.8	Other Alzheimer's disease	Chronic	
Mental and behavioural disorders	G30.9	Alzheimer's disease, unspecified	Chronic	
Mental and behavioural disorders	G31.0	Circumscribed brain atrophy	Chronic	
Mental and behavioural disorders	G31.1	Senile degeneration of brain, not elsewhere classified	Chronic	
Mental and behavioural disorders	G31.8	Other specified degenerative diseases of nervous system	Chronic	
Mental and behavioural disorders	F05.1	Delirium superimposed on dementia	Acute	
Mental and behavioural disorders	F10.7	Mental and behavioural disorders due to use of alcohol - Residual and late-onset psychotic disorder	Chronic	
Nutritional deficiencies	E40	Kwashiorkor		Non-incentivized
Nutritional deficiencies	E41	Nutritional marasmus		Non-incentivized
Nutritional deficiencies	E42	Marasmic kwashiorkor		Non-incentivized
Nutritional deficiencies	E43	Unspecified severe protein-energy malnutrition		Non-incentivized
Nutritional deficiencies	E55.0	Rickets, active		Non-incentivized
Nutritional deficiencies	E64.3	Sequelae of rickets		Non-incentivized
Nutritional, endocrine and metabolic	E11.0–E11.8	Non-insulin-dependent diabetes mellitus	Chronic	Incentivized
Nutritional, endocrine and metabolic	E12	Malnutrition-related diabetes mellitus	Chronic	
Nutritional, endocrine and metabolic	E13.0–E13.8	Other specified diabetes mellitus	Chronic	Incentivized
Nutritional, endocrine and metabolic	E14.0–E14.8	Unspecified diabetes mellitus	Chronic	Incentivized
Other vaccine preventable	A35	Other tetanus		Non-incentivized
Other vaccine preventable	A36	Diphtheria	Acute	Non-incentivized
Other vaccine preventable	A37	Whooping cough	Acute	Non-incentivized
Other vaccine preventable	A80	Acute poliomyelitis		Non-incentivized
Other vaccine preventable	B05	Measles	Acute	Non-incentivized
Other vaccine preventable	B06	Rubella [German measles]	Acute	Non-incentivized
Other vaccine preventable	B16.1	Acute hepatitis B with delta-agent (coinfection) without hepatic coma	Acute	Non-incentivized
Other vaccine preventable	B16.9	Acute hepatitis B without delta-agent and without hepatic coma	Acute	Non-incentivized
Other vaccine preventable	B18.0	Chronic viral hepatitis B with delta-agent	Chronic	Non-incentivized
Other vaccine preventable	B18.1	Chronic viral hepatitis B without delta-agent	Chronic	Non-incentivized
Other vaccine preventable	B26	Mumps		Non-incentivized
Other vaccine preventable	G00.0	Haemophilus meningitis		Non-incentivized
Other vaccine preventable	M01.4	Rubella arthritis	Acute	Non-incentivized
Pelvic inflammatory disease	N70	Salpingitis and oophoritis		Non-incentivized
Pelvic inflammatory disease	N73	Other female pelvic inflammatory diseases		Non-incentivized

Disease group	ICD10 code	ICD 10 Name	NHS Outcomes Framework 2014/2015	Harrison et al 2013
Pelvic inflammatory disease	N74	Female pelvic inflammatory disorders in diseases classified elsewhere		Non-incentivized
Perforated/bleeding ulcer	K20	Oesophagitis	Acute	
Perforated/bleeding ulcer	K21	Gastro-oesophageal reflux disease	Acute	
Perforated/bleeding ulcer	K25.0–K25.2	Gastric ulcer	Acute	Non-incentivized
Perforated/bleeding ulcer	K25.4–K25.6	Gastric ulcer	Acute	Non-incentivized
Perforated/bleeding ulcer	K26.0–K26.2	Duodenal ulcer	Acute	Non-incentivized
Perforated/bleeding ulcer	K26.4–K26.6	Duodenal ulcer	Acute	Non-incentivized
Perforated/bleeding ulcer	K27.0–K27.2	Peptic ulcer, site unspecified	Acute	Non-incentivized
Perforated/bleeding ulcer	K27.4–K27.6	Peptic ulcer, site unspecified	Acute	Non-incentivized
Perforated/bleeding ulcer	K28.0–28.2	Gastrojejunal ulcer	Acute	Non-incentivized
Perforated/bleeding ulcer	K28.4–K28.6	Gastrojejunal ulcer	Acute	Non-incentivized
Pyelonephritis and kidney/urinary tract infections	N10	Acute tubulo-interstitial nephritis	Acute	Non-incentivized
Pyelonephritis and kidney/urinary tract infections	N11	Chronic tubulo-interstitial nephritis	Acute	Non-incentivized
Pyelonephritis and kidney/urinary tract infections	N12	Tubulo-interstitial nephritis, not specified as acute or chronic	Acute	Non-incentivized
Pyelonephritis and kidney/urinary tract infections	N13.6	Pyonephrosis	Acute	Non-incentivized
Pyelonephritis and kidney/urinary tract infections	N15.9	Renal tubulo-interstitial disease, unspecified	Acute	
Pyelonephritis and kidney/urinary tract infections	N30.0	Acute cystitis	Acute	Non-incentivized
Pyelonephritis and kidney/urinary tract infections	N30.8	Other cystitis	Acute	Non-incentivized
Pyelonephritis and kidney/urinary tract infections	N30.9	Cystitis, unspecified	Acute	Non-incentivized
Pyelonephritis and kidney/urinary tract infections	N39.0	Urinary tract infection, site not specified	Acute	
Stroke	I61	Intracerebral haemorrhage		Incentivized
Stroke	I62	Other nontraumatic intracranial haemorrhage		Incentivized
Stroke	I63	Cerebral infarction		Incentivized
Stroke	I64	Stroke, not specified as haemorrhage or infarction		Incentivized
Stroke	I66	Occlusion and stenosis of cerebral arteries, not resulting in cerebral infarction		Incentivized
Stroke	I67.2	Cerebral atherosclerosis		Incentivized
Stroke	I69.8	Sequelae of other and unspecified cerebrovascular diseases		Incentivized
Stroke	R47.0	Dysphasia and aphasia		Incentivized

Note. The set of codes defining All ACSCs is the union of sets of codes defining chronic and acute ACSC [13] and incentivised and non-incentivise ACSCs [14]. Incentivised ACSCs are those whose care was incentivised under the QOF in all years 2004 to 2017.

Table A3: Number and annual growth rate of ACSC emergency admissions

	All ACSCs		Incentivised	
	N	Growth rate	N	Growth rate
**2004**	1955617		1111378	
**2005**	2004987	2.52%	1098023	-1.20%
**2006**	2034383	1.47%	1088389	-0.88%
**2007**	1947115	-4.29%	1012077	-7.01%
**2008**	2119358	8.85%	1086936	7.40%
**2009**	2193660	3.51%	1076740	-0.94%
**2010**	2303279	5.00%	1104581	2.59%
**2011**	2329548	1.14%	1098469	-0.55%
**2012**	2460668	5.63%	1135639	3.38%
**2013**	2490974	1.23%	1127694	-0.70%
**2014**	2427684	-2.54%	1145161	1.55%
**2015**	2523981	3.97%	1169832	2.15%
**2016**	2498565	-1.01%	1060092	-9.38%
**2017**	2508552	0.40%	1088585	2.69%
**2004 to 2017**		**28.27%**		**-2.05%**

Note. See Table A5 for a list of ICD10 codes for ACSCs. As with other studies[2,3] we found that 2007 (financial year 2007/8) was peculiar in that the number of ACSCs fell by 4.3%. This may be a result of changes in coding following the roll out of a prospective pricing regime for hospitals which linked payment to the number (and type) cases treated. There was an anomalously large fall in ACSCs classified as non-incentivised using the definitions in Harrison et al. (2014)[15] in 2014 (financial year 2014/15).

Table A4 : Yearly average local correlation of ISARs

	Global Index
2004	0.527
2005	0.500
2006	0.527
2007	0.576
2008	0.606
2009	0.576
2010	0.596
2011	0.572
2012	0.570
2013	0.536
2014	0.596
2015	0.612
2016	0.627
2017	0.446

*Note. ISAR*s: ACSC admissions indirectly standardised by age and gender. Moran's Global I is a measure of the average degree of correlation of a practice's ISAR with those of local practices. It was calculated using a 5 nearest neighbours row standardised weight matrix. The statistics are significant ($p \leq 0.0001$) in every year. Results using other spatial weight matrices are similar.

Table A5: Clustering of ISARs 2004 – 2017

Year	Spatial clusters	Practices	%	Mean	SD	min	max
2004	HH	722	8.82%	172.21	36.94	114.85	550.62
2004	LL	309	3.77%	48.14	10.97	3.95	72.13
2004	n.s.	7157	87.41%	94.83	28.47	0	367.22
2005	HH	746	9.21%	171.79	33.61	118.62	564.09
2005	LL	378	4.66%	46.14	12.82	5.59	72.84
2005	n.s.	6979	86.13%	96.73	27.23	2.53	316.07
2006	HH	768	9.52%	173.75	35.41	123.19	501.43
2006	LL	381	4.72%	45.79	11.73	7.21	71.22
2006	n.s.	6918	85.76%	95.92	27.36	0	269.55
2007	HH	750	9.37%	164.68	33.63	112.45	419.83
2007	LL	586	7.32%	43	10.6	10.75	68.45
2007	n.s.	6671	83.31%	92.24	26.41	0	234.44
2008	HH	783	9.82%	175.39	34.95	120.45	489.84
2008	LL	581	7.29%	38.99	12.5	5.19	74.18
2008	n.s.	6611	82.90%	98.33	27.73	2.72	243.53
2009	HH	756	9.53%	176.98	38.59	125.94	629.06
2009	LL	583	7.35%	39.48	13.57	7.94	73.17
2009	n.s.	6590	83.11%	100.68	27.31	12.21	289.38
2010	HH	807	10.15%	183.19	38.89	132.05	721.78
2010	LL	612	7.70%	44.25	14.03	8.26	75.74
2010	n.s.	6531	82.15%	103.67	28.22	13.44	294.17
2011	HH	768	9.76%	178	36.47	124.5	557.78
2011	LL	552	7.01%	46.91	13.33	8.59	76.13
2011	n.s.	6552	83.23%	102.93	27.77	19.46	292.91
2012	HH	762	9.71%	185.05	37.95	131.27	610.57
2012	LL	541	6.89%	48.68	13.42	9.99	79.63
2012	n.s.	6545	83.40%	107.25	28.75	25.17	325.24
2013	HH	673	8.67%	184.94	37.82	131.76	625.98
2013	LL	471	6.07%	47.6	14.47	12.49	79.89
2013	n.s.	6615	85.26%	106.93	30.62	25.23	962.19
2014	HH	712	9.40%	176.11	31.14	122.31	360.88
2014	LL	532	7.03%	38.68	13.8	8.28	75.21
2014	n.s.	6328	83.57%	101.74	29.8	12.17	881.62
2015	HH	702	9.51%	178.88	30.08	117.09	341.99
2015	LL	475	6.44%	32.8	16.02	0	71.57
2015	n.s.	6201	84.05%	103.08	29.92	14.38	887.25
2016	HH	723	9.91%	173.47	31.74	123.46	450.75
2016	LL	519	7.11%	33.03	14.94	4.18	66.13
2016	n.s.	6057	82.98%	99.25	28.53	12.21	675.46
2017	HH	576	7.85%	179.73	40.21	118.18	558.96
2017	LL	296	4.03%	31.24	13.81	1.68	60.88
2017	n.s.	6468	88.12%	97.09	34.2	0	954.53

ISARs: ACSC admissions indirectly standardised age and gender. Local clusters are identified using Moran's Local Index of Spatial Association. ns: LISA for practice is not statistically significant at 1%.

Santos R, *et al. BMJ Open* 2020; 10:e039910. doi: 10.1136/bmjopen-2020-039910

Table A6: Transition probabilities (%) between spatial cluster between 2004 and 2017

		Type of cluster in 2017			
		LL	n.s.	HH	Total
Type of cluster in 2004	LL	69.28	30.69	0.03	100
	n.s.	2.4	94.34	3.26	100
	HH	0.06	29.92	70.02	100

Note. n.s. local clustering not significant.

Supplementary Figures

Figure A1 – Practice level ISAR ACSC emergency admission 2017 for stroke, for congestive heart failure, and for flu and pneumonia

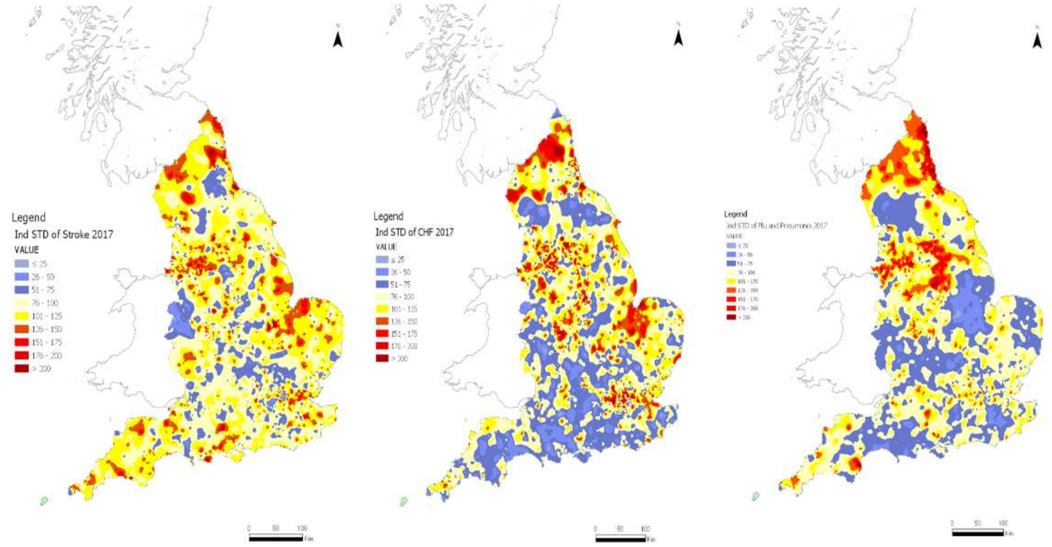

Note: ACSC rates are indirectly standardised by age and gender with expected rates for the reference population computed from 2017 data.

Santos R, *et al. BMJ Open* 2020; 10:e039910. doi: 10.1136/bmjopen-2020-039910

Figure A2 – Change in ACSC ISARs 2004 to 2017

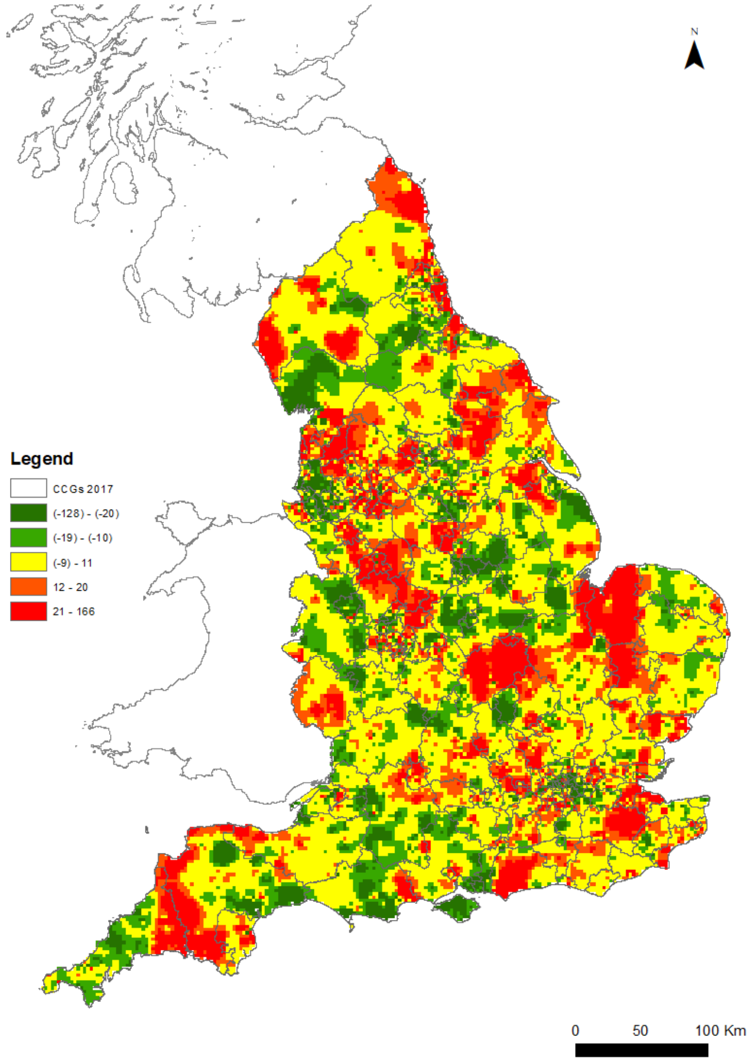

Note. ACSC emergency admissions are indirectly standardised by age and gender with expected rates for the reference population computed from data for all practices for all years 2004 to 2017.

Areas in red indicate increases in admission ratios over the observation period, while areas in green indicate decreases. Some areas with high ACSC ratios in 2004 improved over time, for example areas in and around Liverpool and Hull. Other areas with initial high admission rates did not experience a decrease, for example areas in and around Sunderland and Greater Manchester. Conversely, areas observed to have a relatively low ACSC rates in 2004, for example, Plymouth and York, observed a notable increase to 2017.
