# Supplementary material for: Patterns of emergency admissions for ambulatory care sensitive conditions: a spatial cross-sectional analysis of observational data
Source: BMJ Open. 2020 Nov 4;10(11):e039910. doi: 10.1136/bmjopen-2020-039910 (PMC7643517; doi:10.1136/bmjopen-2020-039910)
Supplement: Author's manuscript [file bmjopen-2020-039910.draft_revisions.pdf]

BMJ Open

BMJ Open is committed to open peer review. As part of this commitment we make the peer review history of every article we publish publicly available.

When an article is published we post the peer reviewers' comments and the authors' responses online. We also post the versions of the paper that were used during peer review. These are the versions that the peer review comments apply to.

The versions of the paper that follow are the versions that were submitted during the peer review process. They are not the versions of record or the final published versions. They should not be cited or distributed as the published version of this manuscript.

BMJ Open is an open access journal and the full, final, typeset and author-corrected version of record of the manuscript is available on our site with no access controls, subscription charges or pay-per-view fees (http://bmjopen.bmj.com).

If you have any questions on BMJ Open's open peer review process please email info.bmjopen@bmj.com

BMJ Open

Patterns of emergency admissions for ambulatory care sensitive conditions: a spatial analysis of observational data

Journal:	*BMJ Open*
Manuscript ID	bmjopen-2020-039910
Article Type:	Original research
Date Submitted by the Author:	04-May-2020
Complete List of Authors:	Santos, Rita; University of York, Centre for Health Economics<br>Rice, Nigel; University of York, Centre for Health Economics; University of York Department of Economics and Related Studies<br>Gravelle, Hugh; University of York, Centre for Health Economics
Keywords:	PRIMARY CARE, HEALTH ECONOMICS, Quality in health care < HEALTH SERVICES ADMINISTRATION & MANAGEMENT

SCHOLARONE™
Manuscripts

I, the Submitting Author has the right to grant and does grant on behalf of all authors of the Work (as defined in the below author licence), an exclusive licence and/or a non-exclusive licence for contributions from authors who are: i) UK Crown employees; ii) where BMJ has agreed a CC-BY licence shall apply, and/or iii) in accordance with the terms applicable for US Federal Government officers or employees acting as part of their official duties; on a worldwide, perpetual, irrevocable, royalty-free basis to BMJ Publishing Group Ltd ("BMJ") its licensees and where the relevant Journal is co-owned by BMJ to the co-owners of the Journal, to publish the Work in this journal and any other BMJ products and to exploit all rights, as set out in our licence.

The Submitting Author accepts and understands that any supply made under these terms is made by BMJ to the Submitting Author unless you are acting as an employee on behalf of your employer or a postgraduate student of an affiliated institution which is paying any applicable article publishing charge ("APC") for Open Access articles. Where the Submitting Author wishes to make the Work available on an Open Access basis (and intends to pay the relevant APC), the terms of reuse of such Open Access shall be governed by a Creative Commons licence – details of these licences and which Creative Commons licence will apply to this Work are set out in our licence referred to above.

Other than as permitted in any relevant BMJ Author's Self Archiving Policies, I confirm this Work has not been accepted for publication elsewhere, is not being considered for publication elsewhere and does not duplicate material already published. I confirm all authors consent to publication of this Work and authorise the granting of this licence.

For peer review only

Patterns of emergency admissions for ambulatory care sensitive conditions: a spatial analysis of observational data

Rita Santos[a] **Nigel Rice**[a,b] **Hugh Gravelle**[a]

[a] Centre for Health Economics, University of York, York, United Kingdom

[b] Department of Economics and Related Studies, University of York, York, United Kingdom

Corresponding author: Rita Santos, rita.santos@york.ac.uk, Centre for Health Economics, Alcuin Block A, University of York, YO10 5DD

Rita Isadora Venâncio dos **Santos**, Centre for Health Economics, University of York, York, United Kingdom
Hugh Stanley Emrys **Gravelle**, Centre for Health Economics, University of York, York, United Kingdom
Nigel Rice, Department of Economics and Related Studies, University of York, York, United Kingdom

MeSH terms: Primary Health Care, Spatial Analysis, Health Care Economics and Organizations, Quality of Health Care, Patient Care.

Word count: 3646

For peer review only

Rita Santos[a] **Nigel Rice**[a,b] **Hugh Gravelle**[a]

[a] Centre for Health Economics, University of York

[b] Department of Economics and Related Studies, University of York

Corresponding author: Rita Santos

Patterns of emergency admissions for ambulatory care sensitive conditions: a spatial analysis of observational data

Abstract

Objectives: To examine the spatial and temporal patterns of English general practices' emergency admissions for Ambulatory Care Sensitive Conditions (ACSC).

Design: Observational study of annual hospital admission data for ACSC emergency admissions at general practice level for all practices in England 2004/5 to 2017/18.

Participants: All patients with an emergency admission to a National Health Service (NHS) hospital in England who were registered with an English GP practice.

Main outcome measure: Practice level age and gender indirectly standardised ratios (ISARs) for emergency admissions for ACSC.

Results: In 2017 41.8% of the total variation in ISARs across practices was *between* the 207 Clinical Commissioning Groups (the administrative unit for general practices) and 58.2% was across practices within CCGs. ACSC ISARs increased by 4.7% between 2004/5 and 2017/18 while those for conditions incentivised by the Quality and Outcomes Framework fell by 20.02%. Practice ISARs are persistent: practices with high rates in 2004/05 also had high rates in 2017/18. Standardising by deprivation as well as age and gender reduced the coefficient of variation of practice ISARs in 2017 by 22%

Conclusions: There is persistent spatial pattern of emergency admissions for ACSC across England both within and across CCGs. We illustrate the reduction in ACSC emergency admissions across the study period for conditions incentivised by the QOF but find that this was not accompanied by a reduction in variation in these admissions across practices. The observed spatial pattern persists when admission rates are standardised by deprivation. The persistence of spatial clusters of high emergency admissions for ACSC within and across CCG

For peer review only

boundaries suggests that policies to reduce potentially unwarranted variation should be targeted at practice level.

Strengths and limitations of this study:

1. This is the first study to explore the spatial pattern of ACSC emergency admissions at GP practice level in England and over a substantial period of time (14 years).
2. We examine the proportion of total variation across practices in ACSC emergency admission that is accounted for by variation between practices within CCGs and variation across CCGs.
3. We consider trends in all ACSC emergency admissions and in those for conditions whose care was incentivised.
4. We examine whether allowing for deprivation in addition to age and gender changes the spatial pattern of ACSC emergency admissions.
5. Identifying how much of the observed variation in ACSC emergency admissions is unwarranted will require practice level data on characteristics of patients to understand how much of the variation is outside the influence of practices and how much is under their control.

For peer review only

Introduction

Ambulatory Care Sensitive Conditions (ACSC) are conditions where good quality primary care can reduce the risk of hospital admission. Rates of emergency hospital admissions for ACSCs are used in many countries as measures of the quality of primary care and geographical variations in them as indicators of inequality.[1,2] Emergency admissions for ACSC are costly - if all Local Authorities (LAs) performed at the level of the best performing quintile of LAs, ACSC emergency admissions would be reduced by 18% with an associated reduction in National Health Service (NHS) expenditure of £238 million.[3]

Although there have been studies of variation across practices in rates of ACSC emergency admissions for specific conditions[4] and of trends over time in ACSC emergency admissions,[5,6] there have been no studies of the geographic variation in overall ACSC emergency admissions across general practices. Blunt et al.[5] show that rates of ACSC emergency admissions standardised by age, gender and deprivation were higher in 2004-2009 for Primary Care Trusts (the then administrative units for general practices) in the north of England compared to the south. NHS Right Care and Public Health England have produced maps of age and gender standardised emergency admission rates for a variety of ambulatory care sensitive conditions at CCG level.[7]

Since ACSC emergency admissions can be reduced by appropriate management in primary care we examine their spatial variation at general practice level. We compare the spatial distributions of age and gender standardised ACSC admissions at general practice level and at the level of clinical commissioning groups (CCG) which are the higher level administrative units to which practices belong. We examine changes in spatial patterns of ACSC admissions across practices from 2004 to 2017, both in total and for ACSC for which care was financially incentivised. We also test for the existence of `hot spots' or clusters of neighbouring practices with similar unusually high (or low) ACSC admission rates which persist over time. Finally, we examine if allowing for practice level differences in deprivation, as well as age and gender, changes the spatial distribution of ACSC admission rates.

For peer review only - http://bmjopen.bmj.com/site/about/guidelines.xhtml

Institutional background

The English National Health Service (NHS) is tax-financed system and free at the point of use (apart from a small charge applied to around 10% of medicines dispensed in primary care). Most general practices are partnerships owned and run by general practitioners. On average they have around 4 GPs, 2 nurses, 1.3 other direct patient care staff, and 8 administrative staff (all staff numbers are full time equivalents) and are responsible for around 7,500 patients.[8] Practices are paid by a mix of lump sum payments, capitation, quality incentive payments, and items of service payments. They are reimbursed for the costs of their premises but have to fund all other expenses, such as the employment of nurses and clerical staff, from their revenue.

In 2004/5 the Quality and Outcomes Framework (QOF) pay for performance scheme was introduced in response to concerns over variation in quality of care provided in general practice. Practices are rewarded for achievement of indicators of clinical quality for a set of chronic conditions and process administrative quality. The QOF accounted for around 15% of practice income in 2004/5[9] and 8% in 2017/18.[10]

Data

We use Hospital Episode Statistics (HES) data on all admissions between 2004/5 and 2017/18 which were coded as an emergency and admitted from a source other than a hospital ward or outpatient clinic. We use the HES patient practice code to attribute emergency admissions to practices by age and gender band. (Supplementary **Table A6** lists data sources.)

There are a variety of definitions of ACSC.[1,11-13] We use a set of ACSCs which is the union of two partially overlapping sets proposed by the NHS Outcomes Framework[13] and Harrison et al.[14] In total we use 178 ICD10 codes (supplementary **Table A5**) for 24 disease groups from the HES primary diagnosis field for patients with an emergency admission.

For each practice we use NHS Digital data on the numbers of patients in 14 age and gender groups. When we standardise ACSC emergency admissions for 2017/18 by deprivation as well as by age and gender we use the Attribution Data Set (NHS Digital) and the Index of Multiple Deprivation (IMD) from ONS. ADS contains the number of practice patients resident in each

For peer review only - http://bmjopen.bmj.com/site/about/guidelines.xhtml
For peer review only

LSOA by age and gender band, while IMD data has an IMD score for each LSOA. From these data we compute the number of patients in 70 age, gender and deprivation quintile groups for each GP practice.

Since very small practices may be new or in the process of merging or closing we include practice-year observations for year *t* only if the practice has more than 1000 patients in years $t-1$, t, and $t+1$. We also exclude outlier practices with more emergency admissions than patients in any age/gender band. In total we excluded 2768 (2.5%) practice-year observations from 1928 practices. The total number of practices included in the analysis fell from 8,188 in 2004 to 7,340 in 2017 reflecting a trend to fewer practices with larger lists.

Practices can have more than one surgery from which they provide care. We obtained data on the location (grid reference from postcodes) of all surgeries of practices from NHS Choices and Connecting for Health archive and current data files: 17,362 surgeries for 2004 and 15,840 in 2017, across 8,188 GP practices.

Methods

Patients and Public Involvement

A Patients and Public Involvement (PPI) group was involved in early discussions of the research topic and in discussions of the methods and presentation of results for a wider audience.

Indirect standardisation

We calculate the indirectly standardised ACSC emergency admissions ratio (ISAR) for practice *i* in year *t* as

$$ISAR_{it} = \frac{Adm_{it}}{ExpAdm_{it}}100$$

where Adm_{it} is the observed number of ACSC emergency admissions in year *t* for practice *i* and $ExpAdm_{it}$ is the expected number of admissions. The latter is the number of admissions practice *i* would have had in year *t* if the age and gender group admission rates of a reference

For peer review only - http://bmjopen.bmj.com/site/about/guidelines.xhtml

population ($RefAdmRate_g$) were applied to practice i's population in those age and gender groups in year t:

$$ExpAdm_{it} = \sum_{g=1}^{14} RefAdmRate_g \times Pop_{igt}$$

When we examine changes in the pattern of ISARs over time (2004 to 2017) we compute the reference population age and gender specific admission rates as the total number of admissions in the respective groups for all practices over the full period 2004 to 2017. The reference population is the number of people in the practices summed across practices and years.:

$$RefAdmRate_g = \left(\sum_{t=2004}^{2017} \sum_i Adm_{igt}\right) / \sum_{t=2004}^{2017} \sum_i Pop_{igt}$$

where Adm_{igt} and Pop_{igt} are admissions and numbers of patients in practice i in age/gender group g in year t. This ensures that changes in practice *ISARs* over time are only due to changes in a practice's age and gender specific admission rates, not to changes in reference admission rates or a practice's age and gender composition.

When we compare the variation in ISARs computed at practice and CCG level for 2017 we use age and gender group admission rates for 2017 to calculate expected admissions. When we standardise by deprivation we use reference groups defined by 2017 age, gender, and deprivation quintile**.**

Spatial pattern analyses

Heat Maps

We attach data on each practice's ISAR to the grid references of all of its surgeries. To depict the spatial pattern of ISARs we impute them to all areas using Inverse Distance Weighting. This interpolation technique creates a smooth surface layer from a finite set of grid references. It is analogous to placing a light sheet over a set of spikes (grid references for surgeries) of different heights (reflecting practice ISARs). The sheet forms contours across the surface of the spikes to give a complete spatial distribution of ISARs. The ISAR imputed for a point is a weighted average of the ISARs of the 12 closest practices with weights $1/d^2$ where d is the distance from the point to the nearest surgery of the practice. Thus the mix of practice ISARs imputed for each point aims to reflect the influence of distance on patient choice of practice.[15]

For peer review only - http://bmjopen.bmj.com/site/about/guidelines.xhtml

For peer review only

Spatial Statistics

Tobler's first law of geography is that "everything is related to everything else, but near things are more related than distant things".[16] In the current context this suggests that a practice's ISAR will be similar to those of nearby practices (nearest five practices): they will be spatially autocorrelated. To test if this holds we use Moran's I statistic[17-21] which measures the average correlation between practices ISARs in year *t* as

$$I_t = \frac{\sum_i \sum_j \omega_{ij} (ISAR_{it} - \overline{ISAR_t})(ISAR_{jt} - \overline{ISAR_t})}{\sum_i (ISAR_{it} - \overline{ISAR_t})^2},$$

where $\overline{ISAR_t}$ is the year *t* mean of $ISAR_{it}$ over all practices and ω_{ij} is a spatial weight based on the minimum straight line distance between surgeries of practices *i* and *j*. We set $\omega_{ij} = 1$ for the five nearest practices and $\omega_{ij} = 0$ otherwise. Positive values of I_t indicate positive spatial autocorrelation.

Moran's I is a global spatial statistic is a measure of the extent to which the spatial pattern over all practices is randomly distributed (as opposed to spatially clustered). To find local clusters of practices with similar ISARs we use a related indicator: Moran's Local Indicator of Spatial Association (LISA)[22]

$$I_{it} = \frac{\left(ISAR_{it} - \overline{ISAR_t}\right)}{n^{-1}\sum_j \left(ISAR_{jt} - \overline{ISAR_t}\right)^2} \sum_j \omega_{ij} \left(ISAR_{jt} - \overline{ISAR_t}\right) .$$

where again we set $\omega_{ij} = 1$ for the five nearest practices and $\omega_{ij} = 0$ otherwise. We use the LISA statistic to identify spatial clusters of practices with similar ISARs. We denote as HH (LL) practices which have above (below) average ISARs and are clustered within a set of nearby practices which also have above (below) average ISARs.

Results

Level of aggregation: CCG vs Practice

Figure 1 displays the spatial pattern of ACSC ISARs in 2017 using data at two levels of aggregation. The left hand map shows the distribution of ISARs (averaged across practices within the CCG) in each of 207 Clinical Commissioning Groups (CCGs). The right hand map has the spatial distribution for the 7,340 individual practices and across 15,840 surgeries. Low

For peer review only - http://bmjopen.bmj.com/site/about/guidelines.xhtml

(under 75) ISAR areas are shaded blue, intermediate (75 to 114) ISAR areas are shaded yellow, and high (125 and above) are shaded red.

< Figure 1 - CCG and practice level ACSC emergency admission 2017 >

The maps show broadly similar spatial patterns, with higher ISARs in the North East, around Liverpool and Manchester, the Midlands around Birmingham, and in parts of the Thames Estuary. However, a comparison across the two maps shows that CCGs with low average ISARs contain areas where practices display high levels of ISARs. We see similar heterogeneity across practices and areas for CCGs that display high levels of ISARs. For example, Northumberland CCG (in the North East) has a moderately high ISAR but the practice level map shows that high ISARs are concentrated in seaside towns and on the border with North Tyneside CCG. Conversely, inland areas have low ISARs. There are also clusters of practices with similar ISARs which span CCG boundaries and differ from the rest of their CCGs.

The CCG maps are based on the average of their respective practice ISARs and accordingly fail to display the nuances of variation at practice level where ACSCs are managed. The coefficient of variation (standard deviation/mean) is 0.30 at CCG level and 0.43 at practice level. More revealingly, 41.8% of the total variance in practice ISARs is between CCGs and 58.2% is due to variation between practices within CCGs. Focusing on CCG level quality metrics is, therefore, likely to lead to an incomplete understanding of local area performance.

Changes over time

< Figure 2 – Change in spatial pattern of ACSC emergency admissions: 2004 vs 2017>

The total number of ACSC emergency admissions increased by 28.3% between 2004/5 and 2017/18 (Supplementary Data Table A1) and the unadjusted ACSC emergency admission rate increased by 11.14%. **Figure 2** compares the spatial pattern of age and gender adjusted ACSC ISARs for 2004 and 2017 using the same reference population (admission rates calculated across all years from 2004 to 2017). (Supplementary Figure 1A maps the change between 2004 and 2017.) The national mean ISAR increased from 95.12 in 2004 to 105.5 in 2013 before declining to 99.6 in 2017 – an increase of 4.7% from 2004 to 2016. The increase in ISARs was

For peer review only - http://bmjopen.bmj.com/site/about/guidelines.xhtml

not uniform. For example, in the North East high ISARs areas became more concentrated in coastal areas. Areas south of the Wash, and along the Thames estuary also displayed increases in ISARs. But in other areas, for example, the Isle of Wight, and the far South West, ACSCs ISARs fell. Overall variation in ISARs, as measured by the coefficient of variation, increased from 0.378 to 0.427 over the period.

Spatial Correlations

ISARs are not randomly distributed geographically across England. Moran's global I index shows statistically significant positive spatial correlation in all years (Appendix **Table A2**): practice ISARs tend to be more similar to those of nearby practices than to practices further away. The Local Indicator of Spatial Association identifies 722 practices in 2004 with high ACSC ISARs which were in clusters of neighbouring practices which also exhibited high ACSC ratios (HH clusters) and 309 practices within spatial clusters displaying low ACSC (LL clusters). The corresponding values in 2017 are 576 and 296 respectively (details in **Table A3).**

Of those practices classified within an HH cluster in 2004, 70% remained in an HH cluster in 2017. Similarly, 69% of practices that were classified within a LL cluster in 2004 were also within a LL cluster in 2017 (**Table A4**). **Figure 3** shows areas that were classified as HH or LL for different lengths of time, with darker shades indicating areas belonging to clusters for longer periods.

<Figure 3 – Persistence of significant spatial cluster for ACSC ISARs emergency admissions from 2004 to 2017>

Practices in the South and South West of England, the Midlands and the along the border with Wales exhibit the most persistent membership of LL clusters. Clusters of persistently high ACSC ratios ("hot spots") are mainly along the North East coast, Barrow-in-Furness, Liverpool, Greater Manchester, South Yorkshire and the West Midlands around Birmingham.

Trends for ACSCs for which care was incentivised

Conditions classified as ambulatory care sensitive are those where better primary care would improve outcomes, including reducing emergency hospitalisations. The Quality and Outcomes

For peer review only - http://bmjopen.bmj.com/site/about/guidelines.xhtml

Framework (QOF) was introduced in 2004 to provide financial incentives linked to indicators of care for some of these conditions. Total *unadjusted* emergency admissions for incentivised ACSC decreased by 2.1% between 2004 and 2017. This compares to an observed increase of 28.3% for all ACSCs. (**Table A1**).

<Figure 4. ACSC for incentivised conditions 2004 and 2017>

Our comparison of trends in ISARs across time allows for changes in the size and age/gender mix of the population. There was a reduction in the year mean age and gender adjusted ISAR for incentivised conditions of 20.8% (112.52 to 89.09) from 2004 to 2017. This compares with an increase in ISAR for all ACSCs over the same period of 4.7% (95.12 to 99.6) These contrasting trends do not prove that the QOF reduced emergency admissions for incentivised ACSCs since they may just be continuations of trends that existed prior to the introduction of the QOF. However, evidence from comparison of pre- and post-QOF does suggest that the QOF did reduce emergency admissions for incentivised ACSCs.[14]

Inspection of the maps in **Figure 4** shows that between 2004 and 2017 there were marked reductions in incentivised ACSC emergency admissions in some areas which previously displayed high ISARs, particularly in the North East and in the Liverpool-Manchester-Leeds-Hull corridor and in the South West. However, areas with initially more moderate ISARs also experienced reductions, for example in Norfolk. The overall dispersion (coefficient of variation) of incentivised ACSC ISARs increased slightly from 0.43 to 0.48 over the period of observation.

Allowing for deprivation

Variations in practice ACSC admission rates which are due to factors outside the control of practices and CCGs are not informative for primary care policy. So far we have allowed for cross-practice variations in age and gender but some of the cross-practice differences are due to variations in other factors not controllable by local policy, such as deprivation[4,14,23]. The right hand map in **Figure 5** shows the 2017 spatial pattern of ACSC ISARs after standardising by deprivation as well as by age and gender (as described in the Methods section) and the left hand map shows the pattern after standardising only by age and gender.

For peer review only - http://bmjopen.bmj.com/site/about/guidelines.xhtml

<Figure 5. Change in ACSC ISAR distribution 2017 after additional standardisation by deprivation >

Additional standardisation by deprivation reduces overall variation in ISARs for emergency ACSC admissions: the coefficient of variation is reduced from 0.43 (left hand map) to 0.36 (right hand map). There is also less overall clustering of practices with similar ISARs: Moran's I falls from 0.45 to 0.39. The number of practices in local clusters with similar ISARs is also reduced: 228 practices (3.1%) are in clusters with high ISARs compared with 576 practices (7.9%) when ratios are standardised only by age and gender. Similarly, the number in clusters with low ISARs is reduced from 296 (4.0%) to 262 to (3.6%).

The reduced variation is illustrated in the maps by the increase in areas shaded yellow which have ISARs relatively close to the mean and the reduction in areas shaded blue or red which have ISARs further from the mean. There are contrasts in the effect of allowing for deprivation: areas along the coast in the North East no longer have high ISARs whereas those on the north Devon coast now have high ISARs. ISARs for parts of Liverpool and Manchester are reduced, whereas some areas in the Midlands have higher ISARs after allowing for deprivation.

Discussion

The mapping of practice ACSC emergency admissions shows that after standardisation by patient age and gender there is considerable spatial variation. Additional standardisation by deprivation reduces this variation somewhat but marked differences across general practices and areas remain. The mapping reveals clusters of practices with similar higher (or lower) than expected standardised ACSC admission rates. These spatial patterns are persistent over a considerable period of time (2004-2017). The mapping demonstrates that emergency admission rates for ACSCs whose care was incentivised by the Quality and Outcomes Framework fell at a faster rate over this period. However, there was little change in the overall variation in emergency ACSC admissions for incentivised conditions.

For peer review only - http://bmjopen.bmj.com/site/about/guidelines.xhtml

Previous studies of the spatial pattern of ACSC emergency admissions have been at higher levels of aggregation and have not examined trends over long periods of time. Our analysis shows that mapping at the level of Clinical Commissioning Groups – the administrative unit for general practice – considerably understates the full extent of variation and does not identify within CCG clusters of practices with similarly high (or low) admission rates and which often span the borders of CCGs.

We found substantial variation in an important outcome for primary care patients that exists after accounting for age and gender. Additionally standardising for deprivation, which is outside the control of practices and CCGs, but can be influenced by national policy, led only to a small reduction in variation. This suggests, though it does not prove, that there is likely to be unwarranted variation after controlling for further additional factors not under the control of practices. Richer data on patients, on practices (staffing, resourcing, and quality) and local environmental factors, combined with multivariate regression modelling, will be required to determine how much practice level variation is unwarranted and, accordingly, how much is potentially amenable to policy.

For peer review only

For peer review only - http://bmjopen.bmj.com/site/about/guidelines.xhtml

Acknowledgements. We are grateful for comments from Tim Doran, Michael Reaker, Sheila Miller, John Gower, Kathleen Murphy, Ann Wands and Patricia Southgate and to Mark Harrison for sharing his code for extraction of ACSCs.

Contributors. RS developed the research questions, undertook data analysis and drafted the paper. HG and NR helped develop the research questions and methods, supervised and commented on the analysis and contributed to drafting the paper. RS is the guarantor.

Funding.
RS was funded in part by NIHR Doctoral Fellowship (DRF 2014-07-055).

Disclaimer. The views expressed are those of the authors and not necessarily those of the funder. The funder had no role in the analysis, interpretation of results, or writing of the manuscript.

Licence declaration. The Corresponding Author has the right to grant on behalf of all authors and does grant on behalf of all authors, an exclusive licence on a worldwide basis to the BMJ Publishing Group Ltd to permit this article (if accepted) to be published in BMJ editions and any other BMJPGL products and sublicences such use and exploit all subsidiary rights, as set out in our licence.

Transparency declaration - RS affirms that this manuscript is an honest, accurate, and transparent account of the study being reported; that no important aspects of the study have been omitted; and that any discrepancies from the study as planned have been explained.

Competing interests. None declared. All authors have completed the ICMJE uniform disclosure form at www.icmje.org/coi_disclosure.pdf and declare: no support from any organisation for the submitted work [or describe if any]; no financial relationships with any organisations that might have an interest in the submitted work in the previous three years; no other relationships or activities that could appear to have influenced the submitted work.

Ethical approval. Ethical approval not required for the use of aggregate practice level data as included in this study.

Data availability statement. Hospital Episode Statistics are Copyright ©2004/05-2016/17 Health and Social Care Information Centre, DARS-NIC 84254-J2G1Q-V2.13, all rights reserved and re-used with the permission of NHS Digital. No additional data available.

Dissemination declaration: the results have been disseminated to the patient involvement group involved in early discussions of the research topic and in discussions of the methods and presentation of results for a wider audience.

ORCID IDs
Rita Santos https://orcid.org/0000-0001-7953-1960
Hugh Gravelle https://orcid.org/0000-0002-7753-4233
Nigel Rice https://orcid.org/0000-0003-0312-823X

For peer review only - http://bmjopen.bmj.com/site/about/guidelines.xhtml

References

1. Purdy S, Griffin T, Salisbury C, Sharp D. Ambulatory care sensitive conditions: terminology and disease coding need to be more specific to aid policy makers and clinicians. Public Health. 2009;123(2):169-73.

2. Blunt T. Focus on preventable admissions: Trends in emergency admissions for ambulatory care sensitive conditions, 2001 to 2013. 2013.

3. Tian Y, Dixon A, Gao H. Emergency hospital admissions for ambulatory care-sensitive conditions: identifying the potential for reductions https://www.kingsfund.org.uk/sites/default/files/field/field_publication_file/data-briefing-emergency-hospital-admissions-for-ambulatory-care-sensitive-conditions-apr-2012.pdf2012 [

4. Dusheiko M, Doran T, Gravelle H, Fullwood C, Roland M. Does Higher Quality of Diabetes Management in Family Practice Reduce Unplanned Hospital Admissions? Health Services Research. 2011;46(1p1):27-46.

5. Blunt T, Bardsley M, Dixon J. Trends in emergency admissions in England 2004–2009: is greater efficiency breeding inefficiency? Research summary report, Nuffield Trust2010.

6. Bardsley M, Blunt I, Davies S, Dixon J. Is secondary preventive care improving? Observational study of 10-year trends in emergency admissions for conditions amenable to ambulatory care. BMJ Open. 2013;3(1).

7. Public Health England. The 2nd Atlas of Variation in NHS Diagnostic Services in England: Reducing unwarranted variation to improve health outcomes and value. 2017.

8. NHS Digital. General and Personal Medical Services, England September 2015 - March 2016, Provisional Experimental statistics. http://content.digital.nhs.uk/catalogue/PUB217722016 [

9. Roland M. Linking Physicians' Pay to the Quality of Care — A Major Experiment in the United Kingdom. New England Journal of Medicine. 2004;351(14):1448-54. PubMed PMID: 15459308.

10. NHS Digital. NHS Payments to General Practice. https://files.digital.nhs.uk/6D/2284F8/nhspaymentsgp-17-18-rep.pdf; 2018.

11. Purdy S, Griffin T, Salisbury C, Sharp D. Prioritizing ambulatory care sensitive hospital admissions in England for research and intervention: a Delphi exercise. Primary Health Care Research & Development. 2010 2010/001/001;11(1):41-50.

12. Coleman P, Nicholl J. Consensus methods to identify a set of potential performance indicators for systems of emergency and urgent care. Journal of Health Services Research & Policy. 2010 April 1, 2010;15(suppl 2):12-8.

13. Department of Health. The NHS Outcomes Framework 2014/15. Technical Appendix. www.gov.uk/dh; 2013.

14. Harrison MJ, Dusheiko M, Sutton M, Gravelle H, Doran T, Roland M. Effect of a national primary care pay for performance scheme on emergency hospital admissions for ambulatory care sensitive conditions: controlled longitudinal study2014 2014-11-11 23:31:20.

15. Santos R, Gravelle H, Propper C. Does Quality Affect Patients' Choice of Doctor? Evidence from England. The Economic Journal. 2017;127(600):445-94.

16. Tobler WR. A Computer Movie Simulating Urban Growth in the Detroit Region. Economic Geography. 1970;46:234-40.

17. Cliff AD, Ord JK. Spatial Processes: Models and Applications: Pion Limited; 1981 1981.

For peer review only

For peer review only - http://bmjopen.bmj.com/site/about/guidelines.xhtml

18. Anselin L. Spatial econometrics : methods and models. Dordrecht ; Boston: Kluwer Academic Publishers; 1988.

19. Tosetti E, Santos R, Moscone F, Arbia G. The Spatial Dimension of Health Systems. Oxford Research Encyclopedias, Economics and Finance; 2018 2018-07-30.

20. Arbia G. A Primer for Spatial Econometrics: With Applications in R. Palgrave Texts in Econometrics.2014.

21. Moran P. Notes on Continuous Stochastic Phenomena. Biometrika. 1950;37(1/2):17-23.

22. Anselin L. Local Indicators of Spatial Association—LISA. Geographical Analysis. 1995;27(2):93-115.

23. O'Cathain A, Knowles E, Maheswaran R, Pearson T, Turner J, Hirst E, et al. A system-wide approach to explaining variation in potentially avoidable emergency admissions: national ecological study. BMJ Quality & Safety. 2014;23(1):47-55.

24. NHS England. The NHS long term plan. Available from: www.longtermplan.nhs.uk; 2019.

For peer review only

For peer review only - http://bmjopen.bmj.com/site/about/guidelines.xhtml

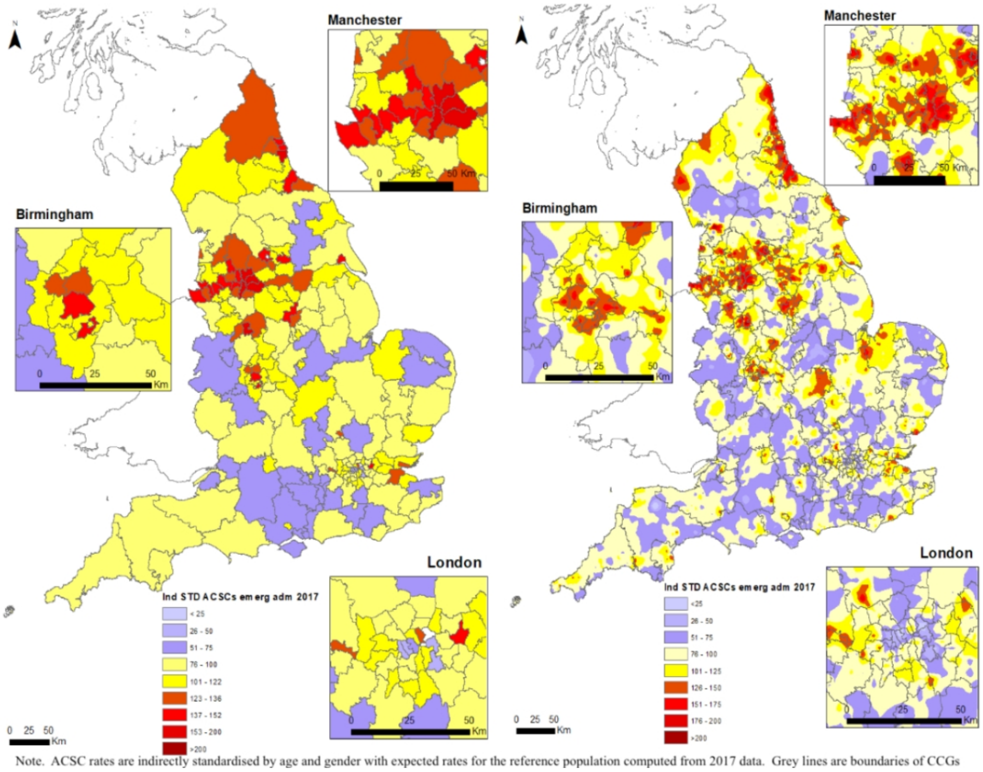

Note. ACSC rates are indirectly standardised by age and gender with expected rates for the reference population computed from 2017 data. Grey lines are boundaries of CCGs

Figure 1 – CCG and practice level ACSC ISARs emergency admission 2017

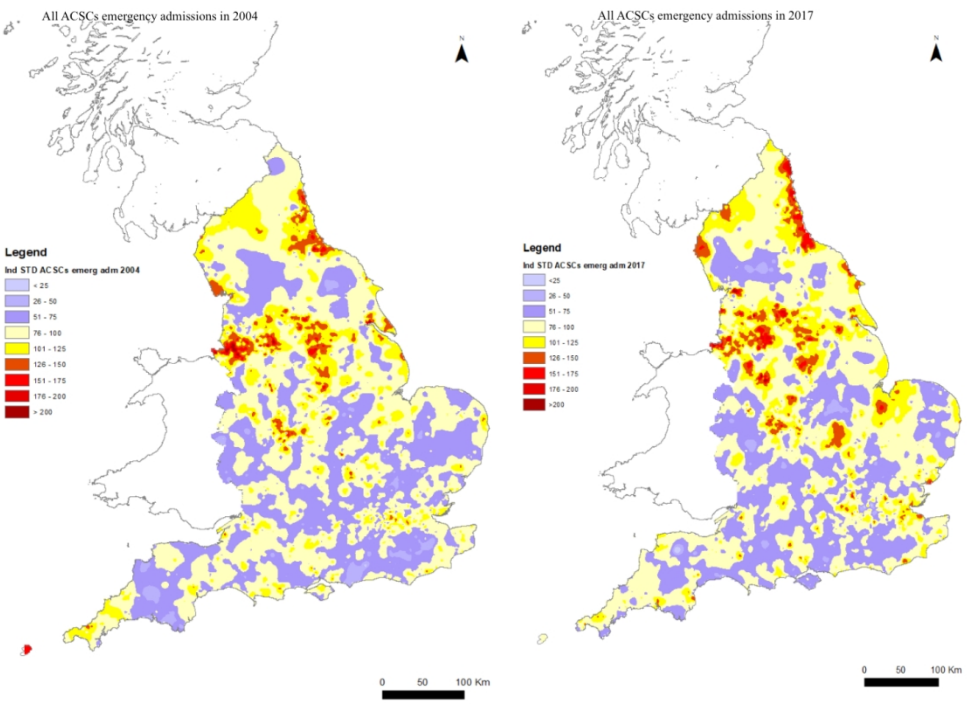

Note. ACSC rates are indirectly standardised by age and gender with expected rates for the reference population computed from data for all practices in all years 2004 to 2017.

Figure 2 - Spatial pattern of ACSC ISARs emergency admissions in 2004, 2017

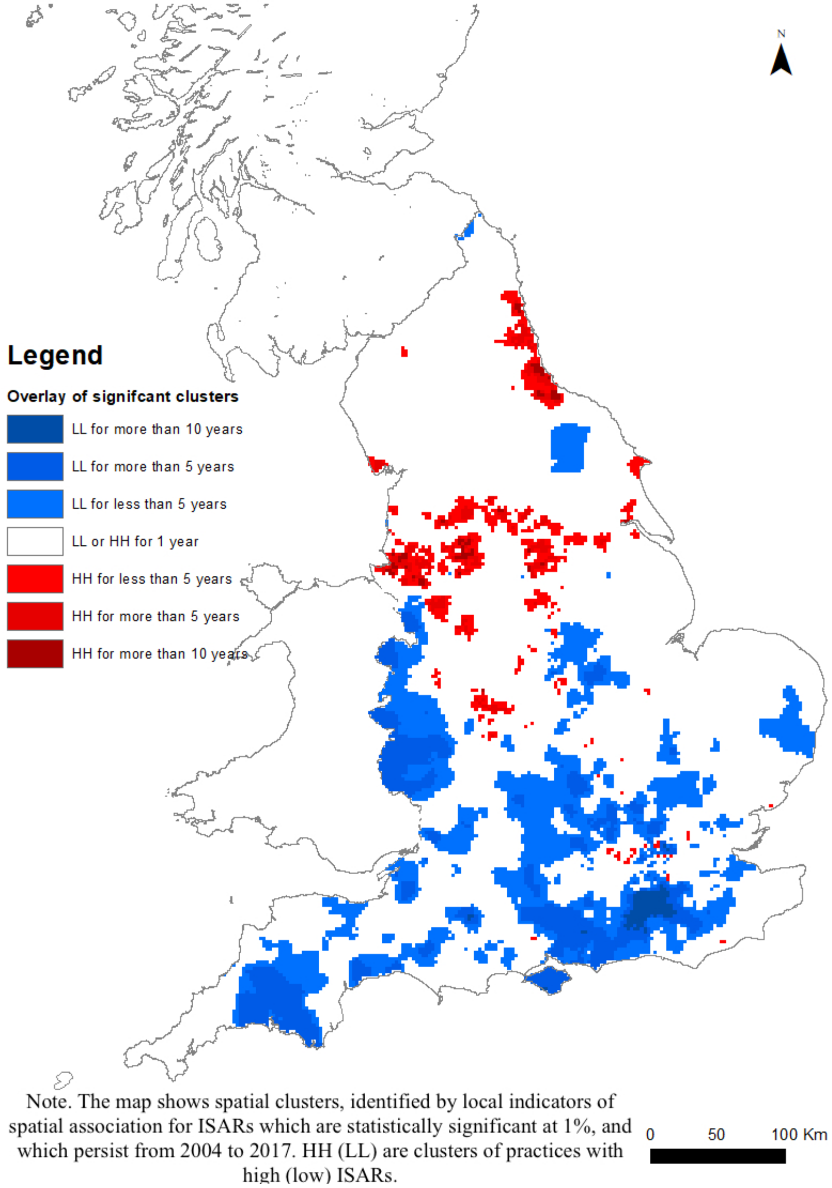

Figure 3 - Persistence of significant spatial cluster for ACSC ISARs emergency admissions from 2004 to 2017

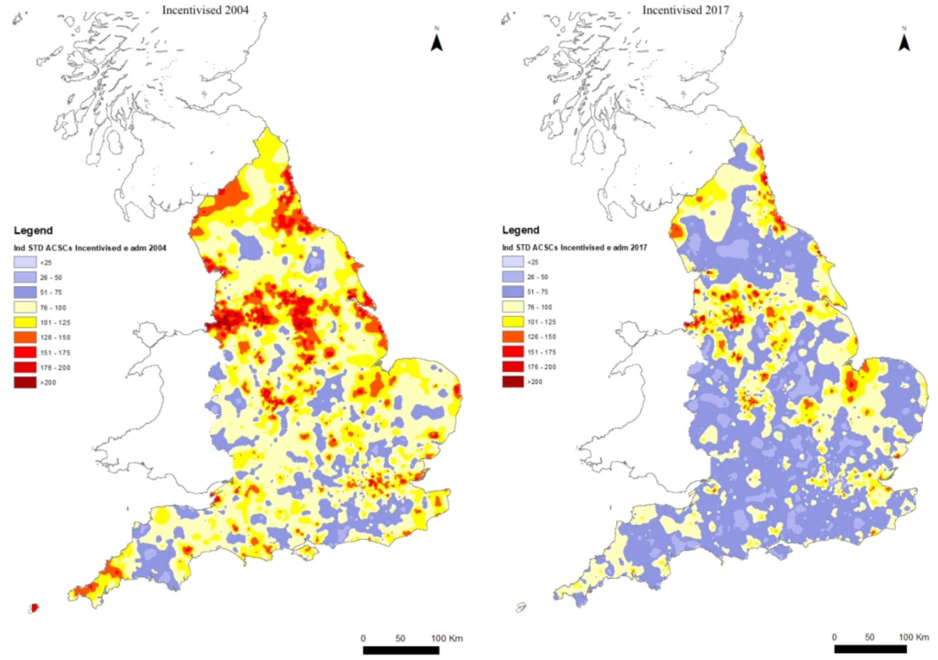

Note. ACSC rates are indirectly standardised by age and gender with expected rates for the reference population computed from data on for all practices in all years 2004 to 2017 for incentivised ACSCs.

Figure 4. Incentivised ACSC ISARs in 2004 and 2017

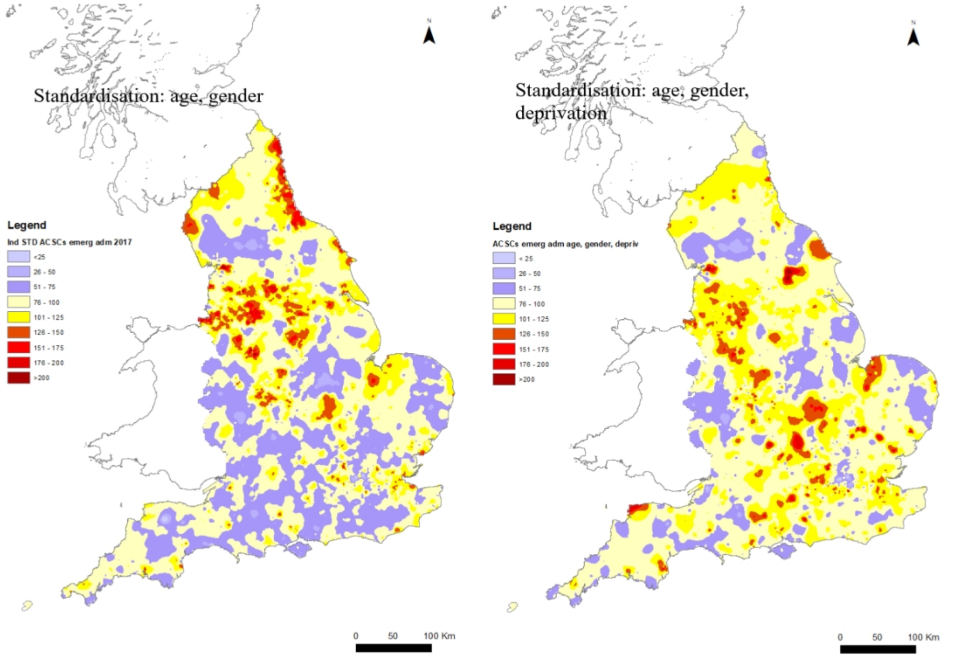

Note. Indirect standardisation. Figures use 2017 data on admissions and practice populations to construct expected number of admissions allowing for age and gender (left hand panel) and for age, gender, and deprivation (right hand panel).

Figure 5. Allowing for deprivation 2017

Supplementary Tables

Table A1. Number and annual growth rate of ACSC emergency admissions 2004-2017
Table A2. Average spatial correlation of ISARs 2004-2017.
Table A3. Clustering of ISARs 2004-2017
Table A4. Transition probabilities between clusters 2004 to 2017.
Table A5. ICD10 codes defining ACSCs
Table A6. Data sources

Table A1: Number and annual growth rate of ACSC emergency admissions

	All ACSCs		Incentivised	
	N	Growth rate	N	Growth rate
**2004**	1955617		1111378	
**2005**	2004987	2.52%	1098023	-1.20%
**2006**	2034383	1.47%	1088389	-0.88%
**2007**	1947115	-4.29%	1012077	-7.01%
**2008**	2119358	8.85%	1086936	7.40%
**2009**	2193660	3.51%	1076740	-0.94%
**2010**	2303279	5.00%	1104581	2.59%
**2011**	2329548	1.14%	1098469	-0.55%
**2012**	2460668	5.63%	1135639	3.38%
**2013**	2490974	1.23%	1127694	-0.70%
**2014**	2427684	-2.54%	1145161	1.55%
**2015**	2523981	3.97%	1169832	2.15%
**2016**	2498565	-1.01%	1060092	-9.38%
**2017**	2508552	0.40%	1088585	2.69%
**2004 to 2017**		**28.27%**		**-2.05%**

Note. See Table A5 for a list of ICD10 codes for ACSCs. As with other studies[2,3] we found that 2007 (financial year 2007/8) was peculiar in that the number of ACSCs fell by 4.3%. This may be a result of changes in coding following the roll out of a prospective pricing regime for hospitals which linked payment to the number (and type) cases treated. There was an anomalously large fall in ACSCs classified as non-incentivised using the definitions in Harrison et al. (2014)[15] in 2014 (financial year 2014/15).

For peer review only

For peer review only - http://bmjopen.bmj.com/site/about/guidelines.xhtml

Table A2 : Yearly average local correlation of ISARs

	Global Index
2004	0.527
2005	0.500
2006	0.527
2007	0.576
2008	0.606
2009	0.576
2010	0.596
2011	0.572
2012	0.570
2013	0.536
2014	0.596
2015	0.612
2016	0.627
2017	0.446

*Note. ISAR*s: ACSC admissions indirectly standardised by age and gender. Moran's Global I is a measure of the average degree of correlation of a practice's ISAR with those of local practices. It was calculated using a 5 nearest neighbours row standardised weight matrix. The statistics are significant ($p \leq 0.0001$) in every year. Results using other spatial weight matrices are similar.

For peer review only

Table A3: Clustering of ISARs 2004 – 2017

Year	Spatial clusters	Practices	%	Mean	SD	min	max
2004	HH	722	8.82%	172.21	36.94	114.85	550.62
2004	LL	309	3.77%	48.14	10.97	3.95	72.13
2004	n.s.	7157	87.41%	94.83	28.47	0	367.22
2005	HH	746	9.21%	171.79	33.61	118.62	564.09
2005	LL	378	4.66%	46.14	12.82	5.59	72.84
2005	n.s.	6979	86.13%	96.73	27.23	2.53	316.07
2006	HH	768	9.52%	173.75	35.41	123.19	501.43
2006	LL	381	4.72%	45.79	11.73	7.21	71.22
2006	n.s.	6918	85.76%	95.92	27.36	0	269.55
2007	HH	750	9.37%	164.68	33.63	112.45	419.83
2007	LL	586	7.32%	43	10.6	10.75	68.45
2007	n.s.	6671	83.31%	92.24	26.41	0	234.44
2008	HH	783	9.82%	175.39	34.95	120.45	489.84
2008	LL	581	7.29%	38.99	12.5	5.19	74.18
2008	n.s.	6611	82.90%	98.33	27.73	2.72	243.53
2009	HH	756	9.53%	176.98	38.59	125.94	629.06
2009	LL	583	7.35%	39.48	13.57	7.94	73.17
2009	n.s.	6590	83.11%	100.68	27.31	12.21	289.38
2010	HH	807	10.15%	183.19	38.89	132.05	721.78
2010	LL	612	7.70%	44.25	14.03	8.26	75.74
2010	n.s.	6531	82.15%	103.67	28.22	13.44	294.17
2011	HH	768	9.76%	178	36.47	124.5	557.78
2011	LL	552	7.01%	46.91	13.33	8.59	76.13
2011	n.s.	6552	83.23%	102.93	27.77	19.46	292.91
2012	HH	762	9.71%	185.05	37.95	131.27	610.57
2012	LL	541	6.89%	48.68	13.42	9.99	79.63
2012	n.s.	6545	83.40%	107.25	28.75	25.17	325.24
2013	HH	673	8.67%	184.94	37.82	131.76	625.98
2013	LL	471	6.07%	47.6	14.47	12.49	79.89
2013	n.s.	6615	85.26%	106.93	30.62	25.23	962.19
2014	HH	712	9.40%	176.11	31.14	122.31	360.88
2014	LL	532	7.03%	38.68	13.8	8.28	75.21
2014	n.s.	6328	83.57%	101.74	29.8	12.17	881.62
2015	HH	702	9.51%	178.88	30.08	117.09	341.99
2015	LL	475	6.44%	32.8	16.02	0	71.57
2015	n.s.	6201	84.05%	103.08	29.92	14.38	887.25
2016	HH	723	9.91%	173.47	31.74	123.46	450.75
2016	LL	519	7.11%	33.03	14.94	4.18	66.13
2016	n.s.	6057	82.98%	99.25	28.53	12.21	675.46
2017	HH	576	7.85%	179.73	40.21	118.18	558.96
2017	LL	296	4.03%	31.24	13.81	1.68	60.88
2017	n.s.	6468	88.12%	97.09	34.2	0	954.53

ISARs: ACSC admissions indirectly standardised age and gender. Local clusters are identified using Moran's Local Index of Spatial Association. ns: LISA for practice is not statistically significant at 1%.

For peer review only

For peer review only - http://bmjopen.bmj.com/site/about/guidelines.xhtml

Table A4: Transition probabilities (%) between spatial cluster between 2004 and 2017

		Type of cluster in 2017			
		LL	n.s.	HH	Total
Type of cluster in 2004	LL	69.28	30.69	0.03	100
	n.s.	2.4	94.34	3.26	100
	HH	0.06	29.92	70.02	100

Note. n.s. local clustering not significant.

For peer review only

For peer review only - http://bmjopen.bmj.com/site/about/guidelines.xhtml

Table A5. ICD10 codes for ACSCs and for incentivised ACSCs.

Disease group	ICD10 code	ICD 10 Name	NHS Outcomes Framework 2014/2015	Harrison et al 2013
Angina	I20	Angina pectoris	Chronic	Incentivized
Angina	I24.0	Coronary thrombosis not resulting in myocardial infarction	Acute	Incentivized
Angina	I24.8	Other forms of acute ischaemic heart disease	Acute	Incentivized
Angina	I24.9	Acute ischaemic heart disease, unspecified	Acute	Incentivized
Asthma	J45	Asthma	Chronic	Incentivized
Asthma	J46	Status asthmaticus	Chronic	Incentivized
Cardiovascular diseases	I13.0	Hypertensive heart and renal disease with (congestive) heart failure	Chronic	Incentivized
Cardiovascular diseases	I25	Chronic ischaemic heart disease	Chronic	Incentivized
Cardiovascular diseases	I48X	Atrial fibrillation and flutter	Chronic	
Cellulitis	L01	Impetigo	Acute	
Cellulitis	L02	Cutaneous abscess, furuncle and carbuncle	Acute	
Cellulitis	L03	Cellulitis	Acute	Non-incentivized
Cellulitis	L04	Acute lymphadenitis	Acute	Non-incentivized
Cellulitis	L08.0	Pyoderma	Acute	Non-incentivized
Cellulitis	L08.8	Other specified local infections of skin and subcutaneous tissue	Acute	Non-incentivized
Cellulitis	L08.9	Local infection of skin and subcutaneous tissue, unspecified	Acute	Non-incentivized
Cellulitis	L88	Pyoderma gangrenosum	Acute	Non-incentivized
Cellulitis	L98.0	Pyogenic granuloma	Acute	Non-incentivized
Cellulitis	I89.1	Lymphangitis	Acute	
Chronic obstructive pulmonary disease	J20	Acute bronchitis	Chronic	Incentivized
Chronic obstructive pulmonary disease	J41	Simple and mucopurulent chronic bronchitis	Chronic	Incentivized
Chronic obstructive pulmonary disease	J42	Unspecified chronic bronchitis	Chronic	Incentivized
Chronic obstructive pulmonary disease	J43	Emphysema	Chronic	Incentivized
Chronic obstructive pulmonary disease	J44	Other chronic obstructive pulmonary disease	Chronic	Incentivized
Chronic obstructive pulmonary disease	J47	Bronchiectasis	Chronic	Incentivized
Congestive heart failure	I11.0	Hypertensive heart disease with (congestive) heart failure	Chronic	Incentivized
Congestive heart failure	I50	Heart failure		Incentivized
Congestive heart failure	J81	Pulmonary oedema	Chronic	Incentivized
Convulsions and epilepsy	G40	Epilepsy	Chronic	Incentivized
Convulsions and epilepsy	G41	Status epilepticus	Chronic	Incentivized
Dehydration and gastroenteritis	E86	Volume depletion	Acute	Non-incentivized
Dehydration and gastroenteritis	K52.2	Allergic and dietetic gastro-enteritis and colitis		Non-incentivized

Disease group	ICD10 code	ICD 10 Name	NHS Outcomes Framework 2014/2015	Harrison et al 2013
Dehydration and gastroenteritis	K52.8	Other specified non-infective gastro-enteritis and colitis		Non-incentivized
Dehydration and gastroenteritis	K52.9	Non-infective gastro-enteritis and colitis, unspecified		Non-incentivized
Diabetes (hypoglycaemic)	E16.2	Hypoglycaemia, unspecified		Incentivized
Diabetes complications	E10.0–E10.8	Insulin-dependent diabetes mellitus	Chronic	Incentivized
Diseases of the blood	D51	Vitamin B12 deficiency anaemia	Chronic	
Diseases of the blood	D52	Folate deficiency anaemia	Chronic	
Ear, nose and throat infections	H66	Suppurative and unspecified otitis media	Acute	Non-incentivized
Ear, nose and throat infections	H67	Otitis media in diseases classified elsewhere	Acute	Non-incentivized
Ear, nose and throat infections	J02	Acute pharyngitis	Acute	Non-incentivized
Ear, nose and throat infections	J03	Acute tonsillitis	Acute	Non-incentivized
Ear, nose and throat infections	J04	Acute laryngitis and tracheitis	Acute	Non-incentivized
Ear, nose and throat infections	J06	Acute upper respiratory infections of multiple and unspecified sites	Acute	Non-incentivized
Ear, nose and throat infections	J31.2	Chronic pharyngitis	Acute	Non-incentivized
Gangrene	R02	Gangrene, not elsewhere classified		Non-incentivized
Hypertension	I10	Essential (primary) hypertension	Chronic	Incentivized
Hypertension	I11.9	Hypertensive heart disease without (congestive) heart failure	Chronic	Incentivized
Influenza and pneumonia	J10	Influenza due to identified influenza virus	Acute	
Influenza and pneumonia	J11	Influenza, virus not identified	Acute	
Influenza and pneumonia	J13X	Pneumonia due to Streptococcus pneumoniae	Acute	
Influenza and pneumonia	J14	Pneumonia due to Haemophilus influenzae	Acute	
Influenza and pneumonia	J15.3	Pneumonia due to streptococcus, group B	Acute	
Influenza and pneumonia	J15.4	Pneumonia due to other streptococci	Acute	
Influenza and pneumonia	J15.7	Pneumonia due to Mycoplasma pneumoniae	Acute	
Influenza and pneumonia	J15.9	Bacterial pneumonia, unspecified	Acute	
Influenza and pneumonia	J16.8	Pneumonia due to other specified infectious organisms	Acute	
Influenza and pneumonia	J18.1	Lobar pneumonia, unspecified	Acute	
Influenza and pneumonia	J18.8	Other pneumonia, organism unspecified	Acute	
Iron deficiency anaemia	D50.1	Sideropenic dysphagia	Chronic	Non-incentivized
Iron deficiency anaemia	D50.8	Other iron deficiency anaemias	Chronic	Non-incentivized
Iron deficiency anaemia	D50.9	Iron deficiency anaemia, unspecified	Chronic	Non-incentivized
Mental and behavioural disorders	F00	Dementia in Alzheimer's disease	Chronic	
Mental and behavioural disorders	F01	Vascular dementia	Chronic	
Mental and behavioural disorders	F02	Dementia in other diseases classified elsewhere	Chronic	
Mental and behavioural disorders	F03	Unspecified dementia	Chronic	

Disease group	ICD10 code	ICD 10 Name	NHS Outcomes Framework 2014/2015	Harrison et al 2013
Mental and behavioural disorders	G30.0	Alzheimer's disease with early onset	Chronic	
Mental and behavioural disorders	G30.1	Alzheimer's disease with late onset	Chronic	
Mental and behavioural disorders	G30.8	Other Alzheimer's disease	Chronic	
Mental and behavioural disorders	G30.9	Alzheimer's disease, unspecified	Chronic	
Mental and behavioural disorders	G31.0	Circumscribed brain atrophy	Chronic	
Mental and behavioural disorders	G31.1	Senile degeneration of brain, not elsewhere classified	Chronic	
Mental and behavioural disorders	G31.8	Other specified degenerative diseases of nervous system	Chronic	
Mental and behavioural disorders	F05.1	Delirium superimposed on dementia	Acute	
Mental and behavioural disorders	F10.7	Mental and behavioural disorders due to use of alcohol - Residual and late-onset psychotic disorder	Chronic	
Nutritional deficiencies	E40	Kwashiorkor		Non-incentivized
Nutritional deficiencies	E41	Nutritional marasmus		Non-incentivized
Nutritional deficiencies	E42	Marasmic kwashiorkor		Non-incentivized
Nutritional deficiencies	E43	Unspecified severe protein-energy malnutrition		Non-incentivized
Nutritional deficiencies	E55.0	Rickets, active		Non-incentivized
Nutritional deficiencies	E64.3	Sequelae of rickets		Non-incentivized
Nutritional, endocrine and metabolic	E11.0–E11.8	Non-insulin-dependent diabetes mellitus	Chronic	Incentivized
Nutritional, endocrine and metabolic	E12	Malnutrition-related diabetes mellitus	Chronic	
Nutritional, endocrine and metabolic	E13.0–E13.8	Other specified diabetes mellitus	Chronic	Incentivized
Nutritional, endocrine and metabolic	E14.0–E14.8	Unspecified diabetes mellitus	Chronic	Incentivized
Other vaccine preventable	A35	Other tetanus		Non-incentivized
Other vaccine preventable	A36	Diphtheria	Acute	Non-incentivized
Other vaccine preventable	A37	Whooping cough	Acute	Non-incentivized
Other vaccine preventable	A80	Acute poliomyelitis		Non-incentivized
Other vaccine preventable	B05	Measles	Acute	Non-incentivized
Other vaccine preventable	B06	Rubella [German measles]	Acute	Non-incentivized
Other vaccine preventable	B16.1	Acute hepatitis B with delta-agent (coinfection) without hepatic coma	Acute	Non-incentivized
Other vaccine preventable	B16.9	Acute hepatitis B without delta-agent and without hepatic coma	Acute	Non-incentivized
Other vaccine preventable	B18.0	Chronic viral hepatitis B with delta-agent	Chronic	Non-incentivized
Other vaccine preventable	B18.1	Chronic viral hepatitis B without delta-agent	Chronic	Non-incentivized
Other vaccine preventable	B26	Mumps		Non-incentivized
Other vaccine preventable	G00.0	Haemophilus meningitis		Non-incentivized
Other vaccine preventable	M01.4	Rubella arthritis	Acute	Non-incentivized
Pelvic inflammatory disease	N70	Salpingitis and oophoritis		Non-incentivized
Pelvic inflammatory disease	N73	Other female pelvic inflammatory diseases		Non-incentivized

For peer review only - http://bmjopen.bmj.com/site/about/guidelines.xhtml

Disease group	ICD10 code	ICD 10 Name	NHS Outcomes Framework 2014/2015	Harrison et al 2013
Pelvic inflammatory disease	N74	Female pelvic inflammatory disorders in diseases classified elsewhere		Non-incentivized
Perforated/bleeding ulcer	K20	Oesophagitis	Acute	
Perforated/bleeding ulcer	K21	Gastro-oesophageal reflux disease	Acute	
Perforated/bleeding ulcer	K25.0–K25.2	Gastric ulcer	Acute	Non-incentivized
Perforated/bleeding ulcer	K25.4–K25.6	Gastric ulcer	Acute	Non-incentivized
Perforated/bleeding ulcer	K26.0–K26.2	Duodenal ulcer	Acute	Non-incentivized
Perforated/bleeding ulcer	K26.4–K26.6	Duodenal ulcer	Acute	Non-incentivized
Perforated/bleeding ulcer	K27.0–K27.2	Peptic ulcer, site unspecified	Acute	Non-incentivized
Perforated/bleeding ulcer	K27.4–K27.6	Peptic ulcer, site unspecified	Acute	Non-incentivized
Perforated/bleeding ulcer	K28.0–28.2	Gastrojejunal ulcer	Acute	Non-incentivized
Perforated/bleeding ulcer	K28.4–K28.6	Gastrojejunal ulcer	Acute	Non-incentivized
Pyelonephritis and kidney/urinary tract infections	N10	Acute tubulo-interstitial nephritis	Acute	Non-incentivized
Pyelonephritis and kidney/urinary tract infections	N11	Chronic tubulo-interstitial nephritis	Acute	Non-incentivized
Pyelonephritis and kidney/urinary tract infections	N12	Tubulo-interstitial nephritis, not specified as acute or chronic	Acute	Non-incentivized
Pyelonephritis and kidney/urinary tract infections	N13.6	Pyonephrosis	Acute	Non-incentivized
Pyelonephritis and kidney/urinary tract infections	N15.9	Renal tubulo-interstitial disease, unspecified	Acute	
Pyelonephritis and kidney/urinary tract infections	N30.0	Acute cystitis	Acute	Non-incentivized
Pyelonephritis and kidney/urinary tract infections	N30.8	Other cystitis	Acute	Non-incentivized
Pyelonephritis and kidney/urinary tract infections	N30.9	Cystitis, unspecified	Acute	Non-incentivized
Pyelonephritis and kidney/urinary tract infections	N39.0	Urinary tract infection, site not specified	Acute	
Stroke	I61	Intracerebral haemorrhage		Incentivized
Stroke	I62	Other nontraumatic intracranial haemorrhage		Incentivized
Stroke	I63	Cerebral infarction		Incentivized
Stroke	I64	Stroke, not specified as haemorrhage or infarction		Incentivized
Stroke	I66	Occlusion and stenosis of cerebral arteries, not resulting in cerebral infarction		Incentivized
Stroke	I67.2	Cerebral atherosclerosis		Incentivized
Stroke	I69.8	Sequelae of other and unspecified cerebrovascular diseases		Incentivized
Stroke	R47.0	Dysphasia and aphasia		Incentivized

Note. The set of codes defining All ACSCs is the union of sets of codes defining chronic and acute ACSC [13] and incentivised and non-incentivise ACSCs [14]. Incentivised ACSCs are those whose care was incentivised under the QOF in all years 2004 to 2017.

For peer review only - http://bmjopen.bmj.com/site/about/guidelines.xhtml

Table A6. Data sources

Data	Data source
Number of patients by age and gender	NHS Digital<br>http://content.digital.nhs.uk/workforce
2015 Index of Multiple Deprivation from Neighbourhood Statistics	Office for National Statistics<br>http://www.neighbourhood.statistics.gov.uk/dissemination/
Attribution Data Set	NHS Digital<br>http://content.digital.nhs.uk/
2017 CCG boundaries	https://data.gov.uk/

For peer review only

For peer review only - http://bmjopen.bmj.com/site/about/guidelines.xhtml

Supplementary Figures

Figure A1 – Change in ACSC ISARs 2004 to 2017

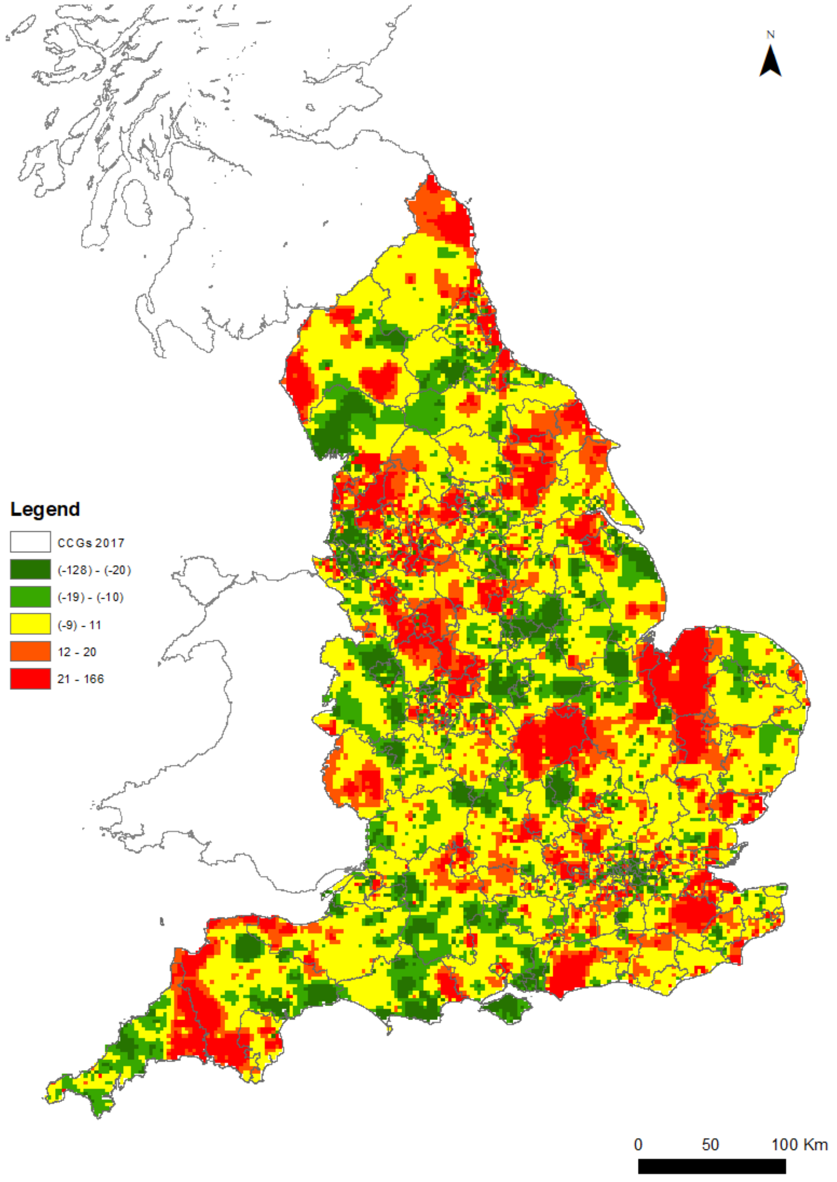

Note. ACSC emergency admissions are indirectly standardised by age and gender with expected rates for the reference population computed from data for all practices for all years 2004 to 2017.

Areas in red indicate increases in admission ratios over the observation period, while areas in green indicate decreases. Some areas with high ACSC ratios in 2004 improved over time, for example areas in and around Liverpool and Hull. Other areas with initial high admission rates did not experience a decrease, for example areas in and around Sunderland and Greater Manchester. Conversely, areas observed to have a relatively low ACSC rates in 2004, for example, Plymouth and York, observed a notable increase to 2017.

For peer review only - http://bmjopen.bmj.com/site/about/guidelines.xhtml

BMJ Open

Patterns of emergency admissions for ambulatory care sensitive conditions: a spatial cross-sectional analysis of observational data

Journal:	*BMJ Open*
Manuscript ID	bmjopen-2020-039910.R1
Article Type:	Original research
Date Submitted by the Author:	19-Sep-2020
Complete List of Authors:	Santos, Rita; University of York, Centre for Health Economics<br>Rice, Nigel; University of York, Centre for Health Economics; University of York Department of Economics and Related Studies<br>Gravelle, Hugh; University of York, Centre for Health Economics
**Primary Subject Heading**:	General practice / Family practice
Secondary Subject Heading:	Health economics
Keywords:	PRIMARY CARE, HEALTH ECONOMICS, Quality in health care < HEALTH SERVICES ADMINISTRATION & MANAGEMENT

SCHOLARONE™
Manuscripts

I, the Submitting Author has the right to grant and does grant on behalf of all authors of the Work (as defined in the below author licence), an exclusive licence and/or a non-exclusive licence for contributions from authors who are: i) UK Crown employees; ii) where BMJ has agreed a CC-BY licence shall apply, and/or iii) in accordance with the terms applicable for US Federal Government officers or employees acting as part of their official duties; on a worldwide, perpetual, irrevocable, royalty-free basis to BMJ Publishing Group Ltd ("BMJ") its licensees and where the relevant Journal is co-owned by BMJ to the co-owners of the Journal, to publish the Work in this journal and any other BMJ products and to exploit all rights, as set out in our licence.

The Submitting Author accepts and understands that any supply made under these terms is made by BMJ to the Submitting Author unless you are acting as an employee on behalf of your employer or a postgraduate student of an affiliated institution which is paying any applicable article publishing charge ("APC") for Open Access articles. Where the Submitting Author wishes to make the Work available on an Open Access basis (and intends to pay the relevant APC), the terms of reuse of such Open Access shall be governed by a Creative Commons licence – details of these licences and which Creative Commons licence will apply to this Work are set out in our licence referred to above.

Other than as permitted in any relevant BMJ Author's Self Archiving Policies, I confirm this Work has not been accepted for publication elsewhere, is not being considered for publication elsewhere and does not duplicate material already published. I confirm all authors consent to publication of this Work and authorise the granting of this licence.

For peer review only

Patterns of emergency admissions for ambulatory care sensitive conditions: a spatial cross-sectional analysis of observational data

Rita Santos[a] **Nigel Rice**[a,b] **Hugh Gravelle**[a]

[a] Centre for Health Economics, University of York, York, United Kingdom

[b] Department of Economics and Related Studies, University of York, York, United Kingdom

Corresponding author: Rita Santos, rita.santos@york.ac.uk, Centre for Health Economics, Alcuin Block A, University of York, YO10 5DD

Rita Isadora Venâncio dos **Santos**, Centre for Health Economics, University of York, York, United Kingdom
Hugh Stanley Emrys **Gravelle**, Centre for Health Economics, University of York, York, United Kingdom
Nigel Rice, Department of Economics and Related Studies, University of York, York, United Kingdom

MeSH terms: Primary Health Care, Spatial Analysis, Health Care Economics and Organizations, Quality of Health Care, Patient Care.

For peer review only

Rita Santos[a] **Nigel Rice**[a,b] **Hugh Gravelle**[a]

[a] Centre for Health Economics, University of York

[b] Department of Economics and Related Studies, University of York

Corresponding author: Rita Santos

Patterns of emergency admissions for ambulatory care sensitive conditions: a spatial cross-sectional analysis of observational data

Abstract

Objectives: To examine the spatial and temporal patterns of English general practices' emergency admissions for Ambulatory Care Sensitive Conditions (ACSC).

Design: Observational study of annual hospital admission data for ACSC emergency admissions at general practice level for all practices in England 2004 to 2017.

Participants: All patients with an emergency admission to a National Health Service (NHS) hospital in England who were registered with an English GP practice.

Main outcome measure: Practice level age and gender indirectly standardised ratios (ISARs) for emergency admissions for ACSC.

Results: In 2017 41.8% of the total variation in ISARs across practices was *between* the 207 Clinical Commissioning Groups (the administrative unit for general practices) and 58.2% was across practices within CCGs. ACSC ISARs increased by 4.7% between 2004 and 2017 while those for conditions incentivised by the Quality and Outcomes Framework fell by 20.02%. Practice ISARs are persistent: practices with high rates in 2004 also had high rates in 2017. Standardising by deprivation as well as age and gender reduced the coefficient of variation of practice ISARs in 2017 by 22%

Conclusions: There is persistent spatial pattern of emergency admissions for ACSC across England both within and across CCGs. We illustrate the reduction in ACSC emergency admissions across the study period for conditions incentivised by the QOF but find that this was not accompanied by a reduction in variation in these admissions across practices. The observed spatial pattern persists when admission rates are standardised by deprivation. The persistence of spatial clusters of high emergency admissions for ACSC within and across CCG

For peer review only - http://bmjopen.bmj.com/site/about/guidelines.xhtml

boundaries suggests that policies to reduce potentially unwarranted variation should be targeted at practice level.

Strengths and limitations of this study:

1. This is the first study to explore the spatial pattern of ACSC emergency admissions at GP practice level in England and over a substantial period of time (14 years) using ACSC emergency admission ratios indirectly standardised by age and gender and also indirectly standardised by age, gender, and deprivation.

2. We use spatial statistical methods to map the geographical distribution of practice ACSC admission and to test for the existence and persistence of clustering of practices with similar admissions.

3. We decompose the total variation in ACSC admissions into variation between practices within administrative areas and variation across administrative areas.

4. We compare changes between 2004 and 2017 in the spatial patterns of ACSCs admissions for conditions whose care was financially incentivised with changes in the patterns of ACSCs for conditions whose care was not incentivised.

5. Understanding how much of the variation in ACSC emergency admissions is outside the influence of practices and how much is potentially amenable to policy requires patient level data.

For peer review only - http://bmjopen.bmj.com/site/about/guidelines.xhtml

Introduction

Ambulatory Care Sensitive Conditions (ACSC) are conditions, such as influenza and pneumonia, diabetes, congestive heart failure, angina, chronic obstructive pulmonary disease, where good quality primary care can reduce the risk of hospital admission. Rates of emergency hospital admissions for ACSCs are used in many countries as measures of the quality of primary care and geographical variations in them as indicators of inequality[1, 2]. Emergency admissions for ACSC are costly - if all Local Authorities (LAs) performed at the level of the best performing quintile of LAs, ACSC emergency admissions would be reduced by 18% with an associated reduction in National Health Service (NHS) expenditure of £238 million[3].

Although there have been studies of variation across practices in rates of ACSC emergency admissions for specific conditions[4] and of trends over time in ACSC emergency admissions,[5,6] there have been no studies of the geographic variation in overall ACSC emergency admissions across general practices. Blunt et al. [5] show that rates of ACSC emergency admissions standardised by age, gender and deprivation were higher in 2004-2009 for Primary Care Trusts (the then administrative units for general practices) in the north of England compared to the south. NHS Right Care and Public Health England have produced maps of age and gender standardised emergency admission rates for a variety of ambulatory care sensitive conditions at Clinical Commissioning Group (CCG) level (the administrative unit to which practices belong.[7]

We make a number of contributions in this study. Since ACSC emergency admissions can be reduced by appropriate management in primary care we examine their spatial variation at general practice level. We use spatially modelling methods to describe the spatial pattern of practice age and gender standardised ACSC emergency admissions in England. We compare the pattern of variation at practice level with that at CCG level. We examine changes in spatial patterns of ACSC admissions across practices from 2004 to 2017, both in total and for ACSCs for which care was financially incentivised via the QOF. We test for the existence of `hot spots' or clusters of neighbouring practices with similar unusually high (or low) ACSC admission rates which persist over time. We examine if allowing for practice level differences in deprivation, as well as age and gender, changes the spatial distribution of ACSC admission rates.

For peer review only - http://bmjopen.bmj.com/site/about/guidelines.xhtml

Institutional background

The English National Health Service (NHS) is tax-financed system and free at the point of use (apart from a small charge applied to around 10% of medicines dispensed in primary care). Most general practices are partnerships owned and run by general practitioners. On average they have around 4 GPs, 2 nurses, 1.3 other direct patient care staff, and 8 administrative staff (all staff numbers are full time equivalents) and are responsible for around 7,500 patients.[8] Practices are paid by a mix of lump sum payments, capitation, quality incentive payments, and items of service payments. They are reimbursed for the costs of their premises but have to fund all other expenses, such as the employment of nurses and clerical staff, from their revenue.

Practices are gatekeepers for outpatient and elective secondary care, though patients have the right to choose any qualified provider in contract with the NHS. For emergency secondary hospital care, patients self refer or are brought in by emergency services, and are almost always admitted via their nearest Accident and Emergency Department (AED).

In 2004/5 the Quality and Outcomes Framework (QOF) pay for performance scheme was introduced in response to concerns over variation in quality of care provided in general practice. Practices are rewarded for achievement of indicators of clinical quality for a set of chronic conditions and process administrative quality. The QOF accounted for around 15% of practice income in 2004[9] and 8% in 2017.[10]

Data

Our data are generally for financial years April 1 to March 31. We use Hospital Episode Statistics (HES) data on all admissions between 2004 and 2017 which were coded as an emergency and admitted from a source other than a hospital ward or outpatient clinic. We use the HES patient practice code to attribute emergency admissions to practices by age and gender band (Supplementary **Table A1** lists data sources).

There are a variety of definitions of ACSC.[1, 11-13] We use a set of ACSCs which is the union of two partially overlapping sets proposed by the NHS Outcomes Framework[13] and Harrison et al.[14] In total we use 178 ICD10 codes (supplementary **Table A2**) for 24 disease groups from

For peer review only
For peer review only - http://bmjopen.bmj.com/site/about/guidelines.xhtml

the HES primary diagnosis field for patients with an emergency admission. This definition is broader than the used in other studies[6, 15], and includes three additional disease groups; mental and behavioural disorders, cardiovascular diseases and stroke, and more ICD 10 codes for some disease groups (for example, N30.0, N30.8 and N30.9 for pyelonephritis and kidney/urinary tract infections). However, our definition excludes vaccine preventable tuberculosis since emergency admissions for this condition are not classified as ACSC in NHS Outcome Framework[13] or Harrison et al.[14] and tuberculosis surveillance is a responsibility of Public Health England.

Management of some ACSCs was financially incentivised by the QOF and to examine changes in these emergency admissions we use the definition of incentivised ACSCs in Harrison et al.[13]

For each practice we use NHS Digital data on the numbers of patients in 14 age and gender groups. When we standardise ACSC emergency admissions for 2017 by deprivation as well as by age and gender we use the Attribution Data Set (NHS Digital) and the Index of Multiple Deprivation (IMD) from ONS. ADS contains the number of practice patients resident in each LSOA by age and gender band, while IMD data has an IMD score for each LSOA. From these data we compute the number of patients in 70 age, gender and deprivation quintile groups for each GP practice.

Since very small practices may be new or in the process of merging or closing we include practice-year observations for year t only if the practice has more than 1000 patients in years $t-1$, t, and $t+1$. We also exclude outlier practices with more emergency admissions than patients in any age/gender band. In total we excluded 2768 (2.5%) practice-year observations from 1928 practices. The total number of practices included in the analysis fell from 8,188 in 2004 to 7,340 in 2017 reflecting a trend to fewer practices with larger lists.

Practices can have more than one surgery from which they provide care. We obtained data on the location (grid reference from postcodes) of all surgeries of practices from NHS Choices and Connecting for Health archive and current data files: 17,362 surgeries for 2004 and 15,840 in 2017, across 8,188 GP practices.

For peer review only

For peer review only - http://bmjopen.bmj.com/site/about/guidelines.xhtml

Methods

Patients and Public Involvement

A Patients and Public Involvement (PPI) group was involved in early discussions of the research topic and in discussions of the methods and presentation of results for a wider audience.

Indirect standardisation

We calculate the indirectly standardised ACSC emergency admissions ratio (ISAR) for practice *i* in year *t* as

$$ISAR_{it} = \frac{Adm_{it}}{ExpAdm_{it}} 100$$

where Adm_{it} is the observed number of ACSC emergency admissions in year *t* for practice *i* and $ExpAdm_{it}$ is the expected number of admissions. The latter is the number of admissions practice *i* would have had in year *t* if the age and gender group admission rates of a reference population ($RefAdmRate_g$) were applied to practice *i*'s population in those age and gender groups in year *t*:

$$ExpAdm_{it} = \sum_{g=1}^{14} RefAdmRate_g \times Pop_{igt}$$

When we examine changes in the pattern of ISARs over time (2004 to 2017) we compute the reference population age and gender specific admission rates as the total number of admissions in the respective groups for all practices over the full period 2004 to 2017. The reference population is the number of people in the practices summed across practices and years.:

$$RefAdmRate_g = \left(\sum_{t=2004}^{2017} \sum_i Adm_{igt}\right) / \sum_{t=2004}^{2017} \sum_i Pop_{igt}$$

where Adm_{igt} and Pop_{igt} are admissions and numbers of patients in practice *i* in age/gender group *g* in year *t*. This ensures that changes in practice *ISARs* over time are only due to changes in a practice's age and gender specific admission rates, not to changes in reference admission rates or a practice's age and gender composition.

When we compare the variation in ISARs computed at practice and CCG level for 2017 we use age and gender group admission rates for 2017 to calculate expected admissions. When we

For peer review only - http://bmjopen.bmj.com/site/about/guidelines.xhtml

standardise by deprivation we use reference groups defined by 2017 age, gender, and deprivation quintile**.**

Spatial pattern analyses

Heat Maps

We attach data on each practice's ISAR to the grid references of all of its surgeries. To depict the spatial pattern of ISARs we impute them to all areas using Inverse Distance Weighting. This interpolation technique creates a smooth surface layer from a finite set of grid references. It is analogous to placing a light sheet over a set of spikes (grid references for surgeries) of different heights (reflecting practice ISARs). The sheet forms contours across the surface of the spikes to give a complete spatial distribution of ISARs. The ISAR imputed for a point is a weighted average of the ISARs of the 12 closest practices with weights $1/d^2$ where d is the distance from the point to the nearest surgery of the practice. Thus the mix of practice ISARs imputed for each point aims to reflect the influence of distance on patient choice of practice.[16]

Spatial Statistics

Tobler's first law of geography is that "everything is related to everything else, but near things are more related than distant things".[17] In the current context this suggests that a practice's ISAR will be similar to those of nearby practices (nearest five practices): they will be spatially autocorrelated. To test if this holds we use Moran's I statistic[18-22] which measures the average correlation between practices ISARs in year t as

$$I_t = \frac{\sum_i \sum_j \omega_{ij}(ISAR_{it} - \overline{ISAR_t})(ISAR_{jt} - \overline{ISAR_t})}{\sum_i (ISAR_{it} - \overline{ISAR_t})^2},$$

where$\overline{ISAR_t}$ is the year t mean of $ISAR_{it}$ over all practices and ω_{ij} is a spatial weight based on the minimum straight-line distance between surgeries of practices i and j. We set $\omega_{ij} = 1$ for the five nearest practices and $\omega_{ij} = 0$ otherwise. This the ISAR for a practice to be compared with the average ISAR of practices that are likely to share the same catchment areas (even in rural areas) and use the same hospital trusts. We set $\omega_{ij} = 1$ for the five nearest practices and $\omega_{ij} = 0$ otherwise. This allows the ISAR for a practice to be compared with the average ISAR

For peer review only - http://bmjopen.bmj.com/site/about/guidelines.xhtml

of practices with overlapping catchment areas and whose patients access the same hospital trusts. Using a distance based threshold could create very large networks for practices in urban areas and much smaller, possibly empty, networks in rural areas.

Positive values of I_t indicate positive spatial autocorrelation.

Moran's I is a global spatial statistic is a measure of the extent to which the spatial pattern over all practices is randomly distributed (as opposed to spatially clustered). To find local clusters of practices with similar ISARs we use a related indicator: Moran's Local Indicator of Spatial Association (LISA)[23]

$$I_{it} = \frac{(ISAR_{it} - \overline{ISAR}_t)}{n^{-1}\sum_j (ISAR_{jt} - \overline{ISAR}_t)^2} \sum_j \omega_{jt}(ISAR_{jt} - \overline{ISAR}_t),$$

where again we set $\omega_{ij} = 1$ for the five nearest practices and $\omega_{ij} = 0$ otherwise. We use the LISA statistic to identify spatial clusters of practices with similar ISARs. We denote as HH (LL) practices which have above (below) average ISARs and are clustered within a set of nearby practices which also have above (below) average ISARs.

Results

Level of aggregation: CCG vs Practice

Figure 1 displays the spatial pattern of ACSC ISARs in 2017 using data at two levels of aggregation. The left-hand map shows the distribution of ISARs (averaged across practices within the CCG) in each of 207 Clinical Commissioning Groups (CCGs). The right hand map has the spatial distribution for the 7,340 individual practices and across 15,840 surgeries. Low (under 75) ISAR areas are shaded blue, intermediate (75 to 114) ISAR areas are shaded yellow, and high (125 and above) are shaded red.

< Figure 1 - CCG and practice level ACSC emergency admission 2017 >

For peer review only - http://bmjopen.bmj.com/site/about/guidelines.xhtml

The maps show broadly similar spatial patterns, with higher ISARs in the North East, around Liverpool and Manchester, the Midlands around Birmingham, and in parts of the Thames Estuary. However, a comparison across the two maps shows that CCGs with low average ISARs contain areas where practices display high levels of ISARs. We see similar heterogeneity across practices and areas for CCGs that display high levels of ISARs. For example, Northumberland CCG (in the North East) has a moderately high ISAR but the practice level map shows that high ISARs are concentrated in seaside towns and on the border with North Tyneside CCG. Conversely, inland areas have low ISARs. There are also clusters of practices with similar ISARs which span CCG boundaries and differ from the rest of their CCGs.

The CCG maps are based on the average of their respective practice ISARs and accordingly fail to display the nuances of variation at practice level where ACSCs are managed. The coefficient of variation (standard deviation/mean) is 0.30 at CCG level and 0.43 at practice level. More revealingly, 41.8% of the total variance in practice ISARs is between CCGs and 58.2% is due to variation between practices within CCGs. Focusing on CCG level quality metrics is, therefore, likely to lead to an incomplete understanding of local area performance.

Our definition of ACSCs includes 24 disease groups with somewhat different spatial patterns. For example, the ISAR's spatial pattern for flu and pneumonia is similar to that for all ACSCs, while there are a higher proportion of practices with high ISARs for CHF and Stroke (Supplementary Figure A1).

Changes over time

< Figure 2 – Change in spatial pattern of ACSC emergency admissions: 2004 vs 2017>

The total number of ACSC emergency admissions increased by 28.3% between 2004/5 and 2017/18 (Supplementary Data **Table A3**) and the unadjusted ACSC emergency admission rate increased by 11.14%. **Figure 2** compares the spatial pattern of age and gender adjusted ACSC ISARs for 2004 and 2017 using the same reference population (admission rates calculated across all years from 2004 to 2017). (Supplementary Figure A2 maps the change between 2004 and 2017.) The national mean ISAR increased from 95.12 in 2004 to 105.5 in 2013 before declining to 99.6 in 2017 – an increase of 4.7% from 2004 to 2016. The increase in ISARs was

For peer review only - http://bmjopen.bmj.com/site/about/guidelines.xhtml

not uniform. For example, in the North East high ISARs areas became more concentrated in coastal areas. Areas south of the Wash, and along the Thames estuary also displayed increases in ISARs. But in other areas, for example, the Isle of Wight, and the far South West, ACSCs ISARs fell. Overall variation in ISARs, as measured by the coefficient of variation, increased from 0.378 to 0.427 over the period.

Spatial Correlations

ISARs are not randomly distributed geographically across England. Moran's global I index shows statistically significant positive spatial correlation in all years (Appendix **Table A4**): practice ISARs tend to be more similar to those of nearby practices than to practices further away. The Local Indicator of Spatial Association identifies 722 practices in 2004 with high ACSC ISARs which were in clusters of neighbouring practices which also exhibited high ACSC ratios (HH clusters) and 309 practices within spatial clusters displaying low ACSC (LL clusters). The corresponding values in 2017 are 576 and 296 respectively (details in **Table A5).**

Of those practices classified within an HH cluster in 2004, 70% remained in an HH cluster in 2017. Similarly, 69% of practices that were classified within a LL cluster in 2004 were also within a LL cluster in 2017 (**Table A6**). **Figure 3** shows areas that were classified as HH or LL for different lengths of time, with darker shades indicating areas belonging to clusters for longer periods.

<Figure 3 – Persistence of significant spatial cluster for ACSC ISARs emergency admissions from 2004 to 2017>

Practices in the South and South West of England, the Midlands and the along the border with Wales exhibit the most persistent membership of LL clusters. Clusters of persistently high ACSC ratios ("hot spots") are mainly along the North East coast, Barrow-in-Furness, Liverpool, Greater Manchester, South Yorkshire and the West Midlands around Birmingham.

Trends for ACSCs for which care was incentivised

Conditions classified as ambulatory care sensitive are those where better primary care would improve outcomes, including reducing emergency hospitalisations. The Quality and Outcomes

For peer review only - http://bmjopen.bmj.com/site/about/guidelines.xhtml

Framework (QOF) was introduced in 2004 to provide financial incentives linked to indicators of care for some of these conditions. Total *unadjusted* emergency admissions for incentivised ACSC decreased by 2.1% between 2004 and 2017. This compares to an observed increase of 28.3% for all ACSCs. (**Table A3**).

<Figure 4. ACSC for incentivised conditions 2004 and 2017>

Our comparison of trends in ISARs across time allows for changes in the size and age/gender mix of the population. There was a reduction in the year mean age and gender adjusted ISAR for incentivised conditions of 20.8% (112.52 to 89.09) from 2004 to 2017. This compares with an increase in ISAR for all ACSCs over the same period of 4.7% (95.12 to 99.6) These contrasting trends do not prove that the QOF reduced emergency admissions for incentivised ACSCs since they may just be continuations of trends that existed prior to the introduction of the QOF. However, evidence from comparison of pre- and post-QOF does suggest that the QOF did reduce emergency admissions for incentivised ACSCs.[14]

Inspection of the maps in **Figure 4** shows that between 2004 and 2017 there were marked reductions in incentivised ACSC emergency admissions in some areas which previously displayed high ISARs, particularly in the North East and in the Liverpool-Manchester-Leeds-Hull corridor and in the South West. However, areas with initially more moderate ISARs also experienced reductions, for example in Norfolk. The overall dispersion (coefficient of variation) of incentivised ACSC ISARs increased slightly from 0.43 to 0.48 over the period of observation.

Allowing for deprivation

Variations in practice ACSC admission rates which are due to factors outside the control of practices and CCGs are not informative for primary care policy. So far we have allowed for cross-practice variations in age and gender but some of the cross-practice differences are due to variations in other factors not controllable by local policy, such as deprivation[4, 14, 24]. **Figure 5** shows the spatial pattern of ACSC ISARs after standardising by deprivation as well as by age and gender (as described in the methods section) for 2004 (left hand panel) and 2017 (right hand panel).

For peer review only - http://bmjopen.bmj.com/site/about/guidelines.xhtml

<Figure 5. Change in ACSC ISAR distribution in 2004 and 2017 after additional standardisation by deprivation >

Variation is reduced after allowing for deprivation. Compared with Figure 2, the maps in Figure 5 which additionally allow for deprivation have more areas shaded yellow, indicating ISARs relatively close to the mean, and fewer areas shaded blue or red, indicating ISARs further from the mean. For 2017 the coefficient of variation is reduced from 0.43 (Figure 2 right hand panel) to 0.36 (Figure 5 right hand panel). For 2004 it is reduced from 0.378 (Figure 2 left hand panel) to 0.28 (Figure 5 left hand map).

Allowing for deprivation also reduces overall clustering of practices with similar ISARs: Moran's I falls from 0.45 to 0.39 in 2017 and from 0.53 to 0.19 in 2004. The number of practices in local clusters with similar ISARs is also reduced by additionally standardising for deprivation, more so in 2004 than in 2017. In 2017 the number of practices in clusters with high ISARs decrease from 576 practices (7.9%) to 228 practices (3.1%). In 2004 the corresponding values are 722 (8.8%) and 238 (3.5%). Similarly, the number in clusters with low ISARs is reduced from 296 (4.0%) to 262 to (3.6%) in 2017 and from 309 (3.8%) to 47 (0.7%).

Allowing for deprivation has different effects in different types of areas. For deprived urban coastal areas, for example in the North East, we no longer observe high ISARs once we standardised for deprivation, whereas less-deprived rural areas, (for example, in the South West) display high ISARs values post standardisation. ISARs for parts of Liverpool and Manchester are reduced, whereas some areas in the Midlands have higher ISARs after allowing for deprivation.

Discussion

For peer review only - http://bmjopen.bmj.com/site/about/guidelines.xhtml

Practice ACSC emergency admissions exhibit considerable spatial variation even after standardisation by patient age and gender. Additional standardisation by deprivation reduces this variation further but marked differences across general practices and areas remain. There are clusters of practices with similar higher (or lower) than expected standardised ACSC admission rates. These spatial patterns persist over a considerable period of time (2004-2017). The spatial analysis also demonstrates, in line with other studies,[13] that emergency admission rates for ACSCs whose care was incentivised by the Quality and Outcomes Framework fell at a faster rate than non-incentivised conditions over the study period. However, there was little change in the overall variation in emergency ACSC admissions for incentivised conditions.

Previous studies of the spatial pattern of ACSC emergency admissions have been undertaken at higher levels of spatial aggregation and have not examined trends over prolonged periods of time. Our analysis shows that mapping at the level of Clinical Commissioning Groups[6] – the administrative unit for general practice – considerably understates the full extent of variation and does not identify within CCG clusters of practices with similarly high (or low) admission rates and which often span the borders of CCGs.

We found substantial variation in an outcome of importance for primary care patients after accounting for age and gender. Additionally, standardising for deprivation, which is outside the control of practices and CCGs, but can be influenced by national policy, reduced observed variation. Allowing for deprivation had different effects in different types of areas (coastal versus inland, urban versus rural), possibly because the deprivation measure is a composite of different types of deprivation which vary across areas and which could have different effects on ACSCs.

The mapping of practice level ACSC emergency admissions standardised for age and gender is a useful method for screening for possible unwarranted variation. But observed variation may be due to factors outside practice control. These include underlying patient morbidity and multi-morbidity, coding practices and admission thresholds in local hospitals, and the provision of community health and social care services by CCGs and local authorities. Richer data on patients, practices (staffing, resourcing, and quality), local services, the mix of hospitals used by patients, and the local environment in which practices operate, combined with multivariate regression modelling, will be required to determine which practices have unduly high ACSCs emergency admissions and how much of the variation across practices is unwarranted and potentially amenable to policy intervention.

For peer review only - http://bmjopen.bmj.com/site/about/guidelines.xhtml

Since 1st July 2019, GP practices in England have been encouraged and funded to collaborate in Primary Care Networks (PCNs) covering populations of 30–50,000 patients[25]. In principle this should reduce variation in outcomes, such as ACSC emergency admission, across practices within PCNs. Its possible effect on variation across PCNs which may adopt different policies is less obvious. The spatial methods employed in this study can be applied to examine variation within and across PCNs.

For peer review only

For peer review only - http://bmjopen.bmj.com/site/about/guidelines.xhtml

Acknowledgements. We are grateful for comments from Tim Doran, Michael Reaker, Sheila Miller, John Gower, Kathleen Murphy, Ann Wands and Patricia Southgate and to Mark Harrison for sharing his code for extraction of ACSCs.

Contributors. RS developed the research questions, undertook data analysis and drafted the paper. HG and NR helped develop the research questions and methods, supervised and commented on the analysis and contributed to drafting the paper. RS is the guarantor.

Funding.
RS was funded in part by NIHR Doctoral Fellowship (DRF 2014-07-055).

Disclaimer. This paper presents independent research funded by the National Institute for Health Research (NIHR). The views expressed are those of the authors and not necessarily those of the NHS, the NIHR or the Department of Health and Social Care.The funder had no role in the analysis, interpretation of results, or writing of the manuscript.

Licence declaration. The Corresponding Author has the right to grant on behalf of all authors and does grant on behalf of all authors, an exclusive licence on a worldwide basis to the BMJ Publishing Group Ltd to permit this article (if accepted) to be published in BMJ editions and any other BMJPGL products and sublicences such use and exploit all subsidiary rights, as set out in our licence.

Transparency declaration - RS affirms that this manuscript is an honest, accurate, and transparent account of the study being reported; that no important aspects of the study have been omitted; and that any discrepancies from the study as planned have been explained.

Competing interests. None declared. All authors have completed the ICMJE uniform disclosure form at www.icmje.org/coi_disclosure.pdf and declare: no support from any organisation for the submitted work [or describe if any]; no financial relationships with any organisations that might have an interest in the submitted work in the previous three years; no other relationships or activities that could appear to have influenced the submitted work.

Ethical approval. Ethical approval not required for the use of aggregate practice level data as included in this study.

Data availability statement. Hospital Episode Statistics are Copyright ©2004/05-2016/17 Health and Social Care Information Centre, DARS-NIC 84254-J2G1Q-V2.13, all rights reserved and re-used with the permission of NHS Digital. No additional data available.

Dissemination declaration: the results have been disseminated to the patient involvement group involved in early discussions of the research topic and in discussions of the methods and presentation of results for a wider audience.

ORCID IDs
Rita Santos https://orcid.org/0000-0001-7953-1960
Hugh Gravelle https://orcid.org/0000-0002-7753-4233
Nigel Rice https://orcid.org/0000-0003-0312-823X

For peer review only - http://bmjopen.bmj.com/site/about/guidelines.xhtml

References

1. Purdy S, Griffin T, Salisbury C, Sharp D. Ambulatory care sensitive conditions: terminology and disease coding need to be more specific to aid policy makers and clinicians. Public Health. 2009;123(2):169-73.

2. Blunt T. Focus on preventable admissions: Trends in emergency admissions for ambulatory care sensitive conditions, 2001 to 2013. 2013.

3. Tian Y, Dixon A, Gao H. Emergency hospital admissions for ambulatory care-sensitive conditions: identifying the potential for reductions https://www.kingsfund.org.uk/sites/default/files/field/field_publication_file/data-briefing-emergency-hospital-admissions-for-ambulatory-care-sensitive-conditions-apr-2012.pdf2012 [

4. Dusheiko M, Doran T, Gravelle H, Fullwood C, Roland M. Does Higher Quality of Diabetes Management in Family Practice Reduce Unplanned Hospital Admissions? Health Services Research. 2011;46(1p1):27-46.

5. Blunt T, Bardsley M, Dixon J. Trends in emergency admissions in England 2004–2009: is greater efficiency breeding inefficiency? Research summary report, Nuffield Trust2010.

6. Bardsley M, Blunt I, Davies S, Dixon J. Is secondary preventive care improving? Observational study of 10-year trends in emergency admissions for conditions amenable to ambulatory care. BMJ Open. 2013;3(1).

7. Public Health England. The 2nd Atlas of Variation in NHS Diagnostic Services in England: Reducing unwarranted variation to improve health outcomes and value. 2017.

8. NHS Digital. General and Personal Medical Services, England September 2015 - March 2016, Provisional Experimental statistics. http://content.digital.nhs.uk/catalogue/PUB217722016 [

9. Roland M. Linking Physicians' Pay to the Quality of Care — A Major Experiment in the United Kingdom. New England Journal of Medicine. 2004;351(14):1448-54. PubMed PMID: 15459308.

10. NHS Digital. NHS Payments to General Practice. https://files.digital.nhs.uk/6D/2284F8/nhspaymentsgp-17-18-rep.pdf; 2018.

11. Purdy S, Griffin T, Salisbury C, Sharp D. Prioritizing ambulatory care sensitive hospital admissions in England for research and intervention: a Delphi exercise. Primary Health Care Research & Development. 2010 2010/001/001;11(1):41-50.

12. Coleman P, Nicholl J. Consensus methods to identify a set of potential performance indicators for systems of emergency and urgent care. Journal of Health Services Research & Policy. 2010 April 1, 2010;15(suppl 2):12-8.

13. Department of Health. The NHS Outcomes Framework 2014/15. Technical Appendix. https://assets.publishing.service.gov.uk/government/uploads/system/uploads/attachment_data/file/256456/NHS_outcomes.pdf; 2013.

14. Harrison MJ, Dusheiko M, Sutton M, Gravelle H, Doran T, Roland M. Effect of a national primary care pay for performance scheme on emergency hospital admissions for ambulatory care sensitive conditions: controlled longitudinal study2014 2014-11-11 23:31:20.

15. Wallace E, McDowell R, Bennett K, Fahey T, Smith SM. Comparison of count-based multimorbidity measures in predicting emergency admission and functional decline in older community-dwelling adults: a prospective cohort study. BMJ Open. 2016;6(9).

16. Santos R, Gravelle H, Propper C. Does Quality Affect Patients' Choice of Doctor? Evidence from England. The Economic Journal. 2017;127(600):445-94.

For peer review only - http://bmjopen.bmj.com/site/about/guidelines.xhtml

17. Tobler WR. A Computer Movie Simulating Urban Growth in the Detroit Region. Economic Geography. 1970;46:234-40.

18. Cliff AD, Ord JK. Spatial Processes: Models and Applications: Pion Limited; 1981 1981.

19. Anselin L. Spatial econometrics : methods and models. Dordrecht ; Boston: Kluwer Academic Publishers; 1988.

20. Tosetti E, Santos R, Moscone F, Arbia G. The Spatial Dimension of Health Systems. Oxford Research Encyclopedias, Economics and Finance; 2018 2018-07-30.

21. Arbia G. A Primer for Spatial Econometrics: With Applications in R. Palgrave Texts in Econometrics.2014.

22. Moran P. Notes on Continuous Stochastic Phenomena. Biometrika. 1950;37(1/2):17-23.

23. Anselin L. Local Indicators of Spatial Association—LISA. Geographical Analysis. 1995;27(2):93-115.

24. O'Cathain A, Knowles E, Maheswaran R, Pearson T, Turner J, Hirst E, et al. A system-wide approach to explaining variation in potentially avoidable emergency admissions: national ecological study. BMJ Quality & Safety. 2014;23(1):47-55.

25. The Kings Fund. Primary care networks explained 2020 [Available from: https://www.kingsfund.org.uk/publications/primary-care-networks-explained.

For peer review only

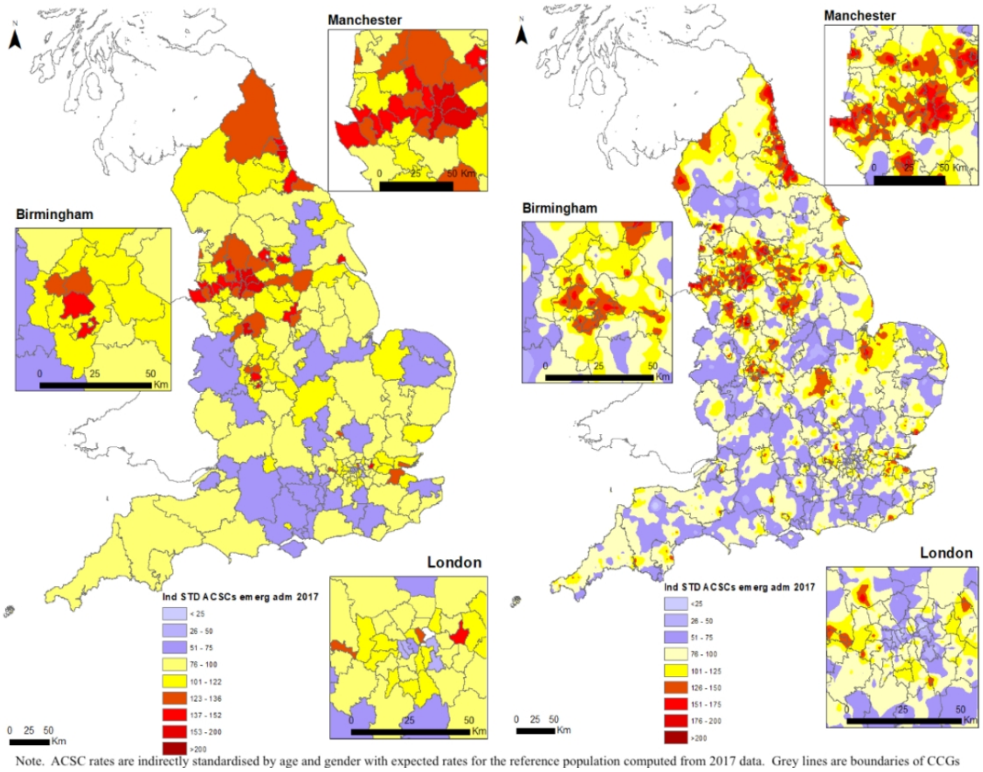

Figure 1 - CCG and practice level ACSC emergency admission 2017

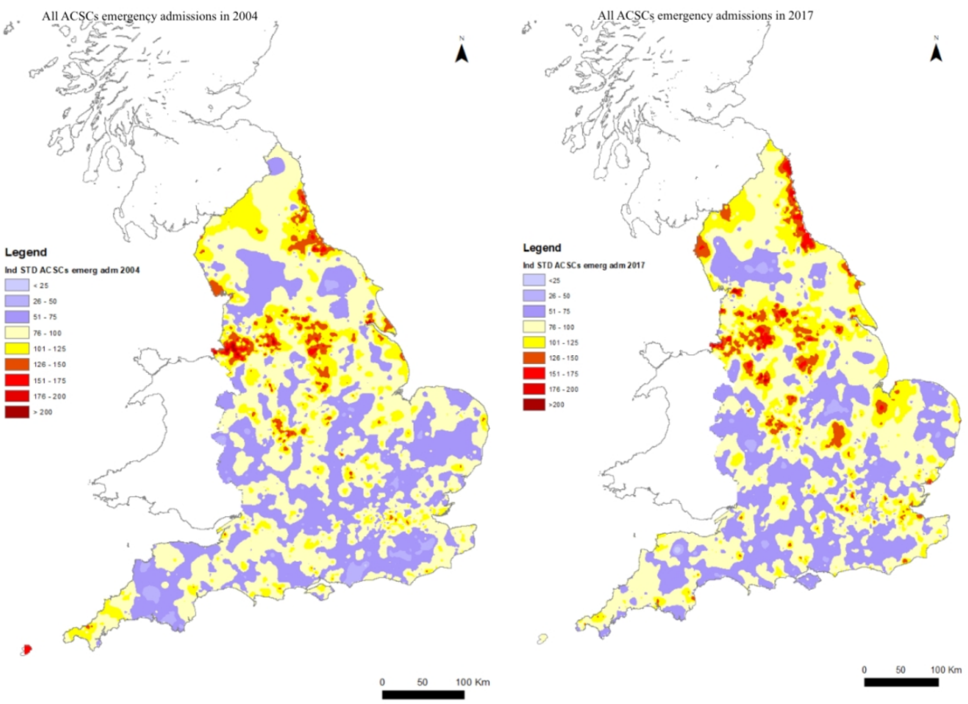

Note. ACSC rates are indirectly standardised by age and gender with expected rates for the reference population computed from data for all practices in all years 2004 to 2017.

Figure 2 – Change in spatial pattern of ACSC emergency admissions: 2004 vs 2017

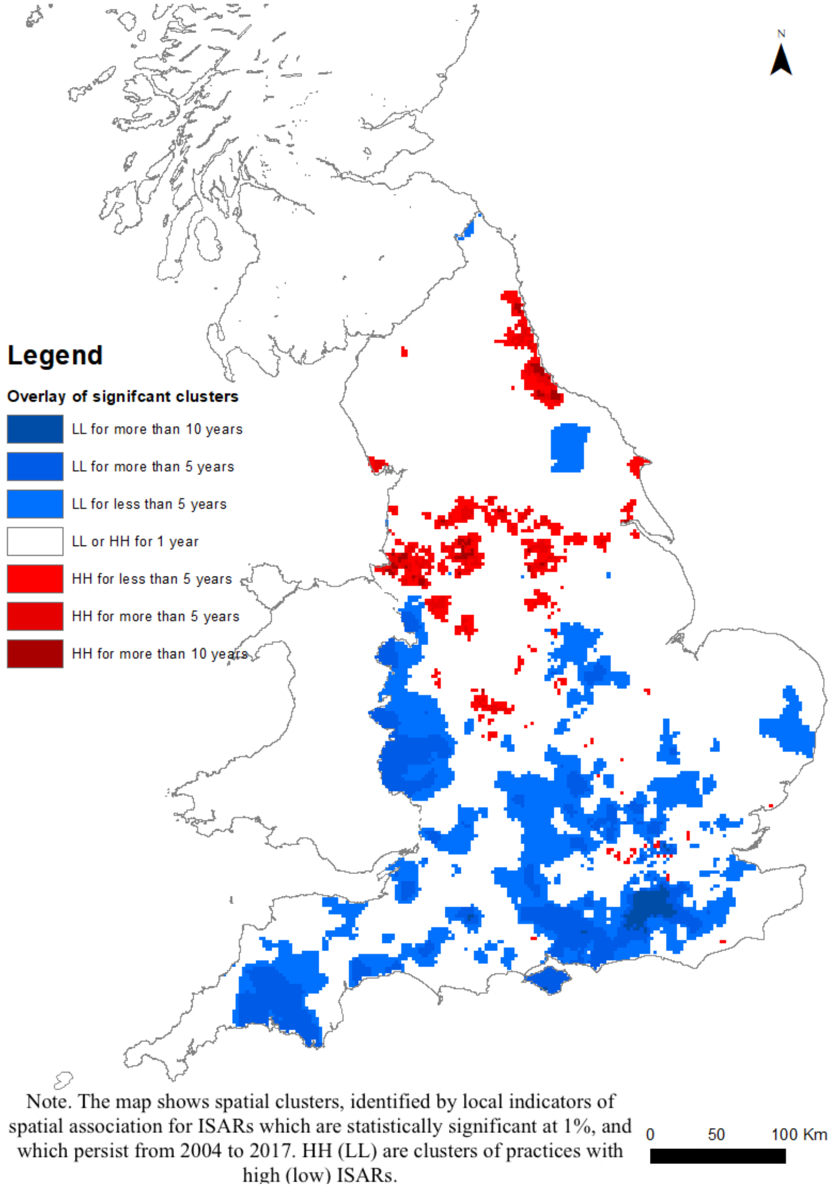

Figure 3 – Persistence of significant spatial cluster for ACSC ISARs emergency admissions from 2004 to 2017

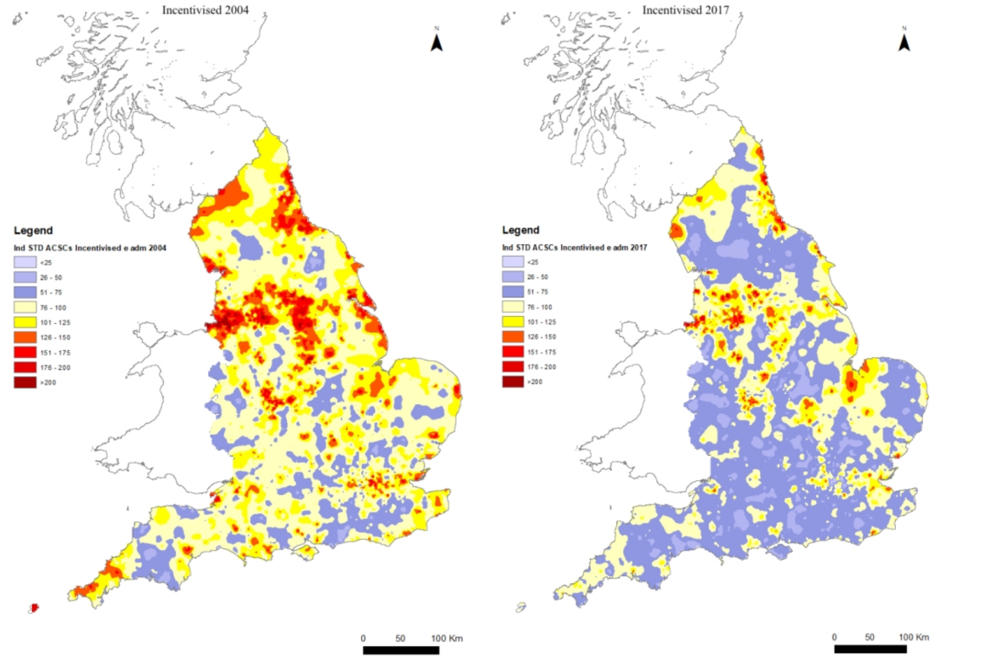

Note. ACSC rates are indirectly standardised by age and gender with expected rates for the reference population computed from data on for all practices in all years 2004 to 2017 for incentivised ACSCs.

Figure 4. ACSC for incentivised conditions 2004 and 2017

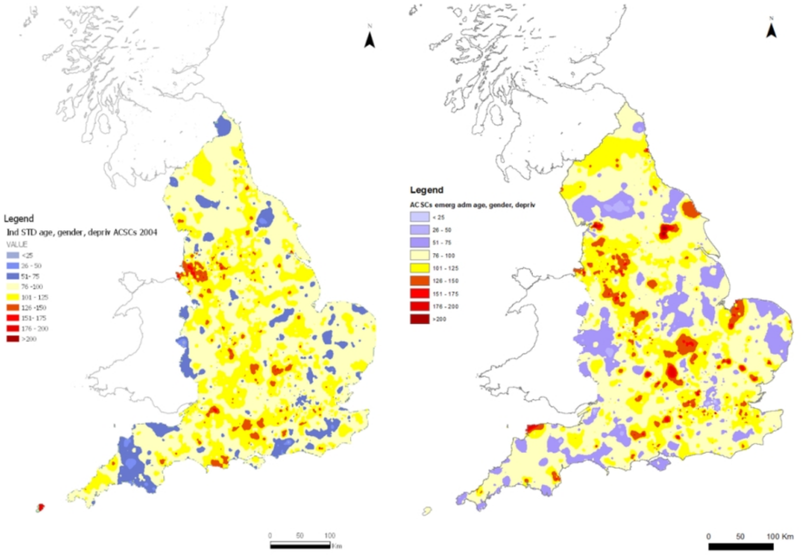

Note. Indirect standardisation. Figures use 2004 (left hand panel) and 2017 (right hand panel) data on admissions and practice populations to construct expected number of admissions allowing for age, gender and deprivation.

Figure 5. Change in ACSC ISAR distribution in 2004 and 2017 after additional standardisation by deprivation

Supplementary Tables

Table A1. Data sources
Table A2. ICD10 codes defining ACSCs
Table A3. Number and annual growth rate of ACSC emergency admissions 2004-2017
Table A4. Average spatial correlation of ISARs 2004-2017.
Table A5. Clustering of ISARs 2004-2017
Table A6. Transition probabilities between clusters 2004 to 2017.

Table A1. Data sources

Data	Data source
Number of patients by age and gender	NHS Digital http://content.digital.nhs.uk/workforce
2015 Index of Multiple Deprivation from Neighbourhood Statistics	Office for National Statistics http://www.neighbourhood.statistics.gov.uk/dissemination/
Attribution Data Set	NHS Digital http://content.digital.nhs.uk/
2017 CCG boundaries	https://data.gov.uk/

For peer review only

Table A2. ICD10 codes for ACSCs and for incentivised ACSCs.

Disease group	ICD10 code	ICD 10 Name	NHS Outcomes Framework 2014/2015	Harrison et al 2013
Angina	I20	Angina pectoris	Chronic	Incentivized
Angina	I24.0	Coronary thrombosis not resulting in myocardial infarction	Acute	Incentivized
Angina	I24.8	Other forms of acute ischaemic heart disease	Acute	Incentivized
Angina	I24.9	Acute ischaemic heart disease, unspecified	Acute	Incentivized
Asthma	J45	Asthma	Chronic	Incentivized
Asthma	J46	Status asthmaticus	Chronic	Incentivized
Cardiovascular diseases	I13.0	Hypertensive heart and renal disease with (congestive) heart failure	Chronic	Incentivized
Cardiovascular diseases	I25	Chronic ischaemic heart disease	Chronic	Incentivized
Cardiovascular diseases	I48X	Atrial fibrillation and flutter	Chronic	
Cellulitis	L01	Impetigo	Acute	
Cellulitis	L02	Cutaneous abscess, furuncle and carbuncle	Acute	
Cellulitis	L03	Cellulitis	Acute	Non-incentivized
Cellulitis	L04	Acute lymphadenitis	Acute	Non-incentivized
Cellulitis	L08.0	Pyoderma	Acute	Non-incentivized
Cellulitis	L08.8	Other specified local infections of skin and subcutaneous tissue	Acute	Non-incentivized
Cellulitis	L08.9	Local infection of skin and subcutaneous tissue, unspecified	Acute	Non-incentivized
Cellulitis	L88	Pyoderma gangrenosum	Acute	Non-incentivized
Cellulitis	L98.0	Pyogenic granuloma	Acute	Non-incentivized
Cellulitis	I89.1	Lymphangitis	Acute	
Chronic obstructive pulmonary disease	J20	Acute bronchitis	Chronic	Incentivized
Chronic obstructive pulmonary disease	J41	Simple and mucopurulent chronic bronchitis	Chronic	Incentivized
Chronic obstructive pulmonary disease	J42	Unspecified chronic bronchitis	Chronic	Incentivized
Chronic obstructive pulmonary disease	J43	Emphysema	Chronic	Incentivized
Chronic obstructive pulmonary disease	J44	Other chronic obstructive pulmonary disease	Chronic	Incentivized
Chronic obstructive pulmonary disease	J47	Bronchiectasis	Chronic	Incentivized
Congestive heart failure	I11.0	Hypertensive heart disease with (congestive) heart failure	Chronic	Incentivized
Congestive heart failure	I50	Heart failure		Incentivized
Congestive heart failure	J81	Pulmonary oedema	Chronic	Incentivized
Convulsions and epilepsy	G40	Epilepsy	Chronic	Incentivized
Convulsions and epilepsy	G41	Status epilepticus	Chronic	Incentivized
Dehydration and gastroenteritis	E86	Volume depletion	Acute	Non-incentivized
Dehydration and gastroenteritis	K52.2	Allergic and dietetic gastro-enteritis and colitis		Non-incentivized

For peer review only - http://bmjopen.bmj.com/site/about/guidelines.xhtml

Disease group	ICD10 code	ICD 10 Name	NHS Outcomes Framework 2014/2015	Harrison et al 2013
Dehydration and gastroenteritis	K52.8	Other specified non-infective gastro-enteritis and colitis		Non-incentivized
Dehydration and gastroenteritis	K52.9	Non-infective gastro-enteritis and colitis, unspecified		Non-incentivized
Diabetes (hypoglycaemic)	E16.2	Hypoglycaemia, unspecified		Incentivized
Diabetes complications	E10.0–E10.8	Insulin-dependent diabetes mellitus	Chronic	Incentivized
Diseases of the blood	D51	Vitamin B12 deficiency anaemia	Chronic	
Diseases of the blood	D52	Folate deficiency anaemia	Chronic	
Ear, nose and throat infections	H66	Suppurative and unspecified otitis media	Acute	Non-incentivized
Ear, nose and throat infections	H67	Otitis media in diseases classified elsewhere	Acute	Non-incentivized
Ear, nose and throat infections	J02	Acute pharyngitis	Acute	Non-incentivized
Ear, nose and throat infections	J03	Acute tonsillitis	Acute	Non-incentivized
Ear, nose and throat infections	J04	Acute laryngitis and tracheitis	Acute	Non-incentivized
Ear, nose and throat infections	J06	Acute upper respiratory infections of multiple and unspecified sites	Acute	Non-incentivized
Ear, nose and throat infections	J31.2	Chronic pharyngitis	Acute	Non-incentivized
Gangrene	R02	Gangrene, not elsewhere classified		Non-incentivized
Hypertension	I10	Essential (primary) hypertension	Chronic	Incentivized
Hypertension	I11.9	Hypertensive heart disease without (congestive) heart failure	Chronic	Incentivized
Influenza and pneumonia	J10	Influenza due to identified influenza virus	Acute	
Influenza and pneumonia	J11	Influenza, virus not identified	Acute	
Influenza and pneumonia	J13X	Pneumonia due to Streptococcus pneumoniae	Acute	
Influenza and pneumonia	J14	Pneumonia due to Haemophilus influenzae	Acute	
Influenza and pneumonia	J15.3	Pneumonia due to streptococcus, group B	Acute	
Influenza and pneumonia	J15.4	Pneumonia due to other streptococci	Acute	
Influenza and pneumonia	J15.7	Pneumonia due to Mycoplasma pneumoniae	Acute	
Influenza and pneumonia	J15.9	Bacterial pneumonia, unspecified	Acute	
Influenza and pneumonia	J16.8	Pneumonia due to other specified infectious organisms	Acute	
Influenza and pneumonia	J18.1	Lobar pneumonia, unspecified	Acute	
Influenza and pneumonia	J18.8	Other pneumonia, organism unspecified	Acute	
Iron deficiency anaemia	D50.1	Sideropenic dysphagia	Chronic	Non-incentivized
Iron deficiency anaemia	D50.8	Other iron deficiency anaemias	Chronic	Non-incentivized
Iron deficiency anaemia	D50.9	Iron deficiency anaemia, unspecified	Chronic	Non-incentivized
Mental and behavioural disorders	F00	Dementia in Alzheimer's disease	Chronic	
Mental and behavioural disorders	F01	Vascular dementia	Chronic	
Mental and behavioural disorders	F02	Dementia in other diseases classified elsewhere	Chronic	
Mental and behavioural disorders	F03	Unspecified dementia	Chronic	

For peer review only - http://bmjopen.bmj.com/site/about/guidelines.xhtml

Disease group	ICD10 code	ICD 10 Name	NHS Outcomes Framework 2014/2015	Harrison et al 2013
Mental and behavioural disorders	G30.0	Alzheimer's disease with early onset	Chronic	
Mental and behavioural disorders	G30.1	Alzheimer's disease with late onset	Chronic	
Mental and behavioural disorders	G30.8	Other Alzheimer's disease	Chronic	
Mental and behavioural disorders	G30.9	Alzheimer's disease, unspecified	Chronic	
Mental and behavioural disorders	G31.0	Circumscribed brain atrophy	Chronic	
Mental and behavioural disorders	G31.1	Senile degeneration of brain, not elsewhere classified	Chronic	
Mental and behavioural disorders	G31.8	Other specified degenerative diseases of nervous system	Chronic	
Mental and behavioural disorders	F05.1	Delirium superimposed on dementia	Acute	
Mental and behavioural disorders	F10.7	Mental and behavioural disorders due to use of alcohol - Residual and late-onset psychotic disorder	Chronic	
Nutritional deficiencies	E40	Kwashiorkor		Non-incentivized
Nutritional deficiencies	E41	Nutritional marasmus		Non-incentivized
Nutritional deficiencies	E42	Marasmic kwashiorkor		Non-incentivized
Nutritional deficiencies	E43	Unspecified severe protein-energy malnutrition		Non-incentivized
Nutritional deficiencies	E55.0	Rickets, active		Non-incentivized
Nutritional deficiencies	E64.3	Sequelae of rickets		Non-incentivized
Nutritional, endocrine and metabolic	E11.0–E11.8	Non-insulin-dependent diabetes mellitus	Chronic	Incentivized
Nutritional, endocrine and metabolic	E12	Malnutrition-related diabetes mellitus	Chronic	
Nutritional, endocrine and metabolic	E13.0–E13.8	Other specified diabetes mellitus	Chronic	Incentivized
Nutritional, endocrine and metabolic	E14.0–E14.8	Unspecified diabetes mellitus	Chronic	Incentivized
Other vaccine preventable	A35	Other tetanus		Non-incentivized
Other vaccine preventable	A36	Diphtheria	Acute	Non-incentivized
Other vaccine preventable	A37	Whooping cough	Acute	Non-incentivized
Other vaccine preventable	A80	Acute poliomyelitis		Non-incentivized
Other vaccine preventable	B05	Measles	Acute	Non-incentivized
Other vaccine preventable	B06	Rubella [German measles]	Acute	Non-incentivized
Other vaccine preventable	B16.1	Acute hepatitis B with delta-agent (coinfection) without hepatic coma	Acute	Non-incentivized
Other vaccine preventable	B16.9	Acute hepatitis B without delta-agent and without hepatic coma	Acute	Non-incentivized
Other vaccine preventable	B18.0	Chronic viral hepatitis B with delta-agent	Chronic	Non-incentivized
Other vaccine preventable	B18.1	Chronic viral hepatitis B without delta-agent	Chronic	Non-incentivized
Other vaccine preventable	B26	Mumps		Non-incentivized
Other vaccine preventable	G00.0	Haemophilus meningitis		Non-incentivized
Other vaccine preventable	M01.4	Rubella arthritis	Acute	Non-incentivized
Pelvic inflammatory disease	N70	Salpingitis and oophoritis		Non-incentivized
Pelvic inflammatory disease	N73	Other female pelvic inflammatory diseases		Non-incentivized

Disease group	ICD10 code	ICD 10 Name	NHS Outcomes Framework 2014/2015	Harrison et al 2013
Pelvic inflammatory disease	N74	Female pelvic inflammatory disorders in diseases classified elsewhere		Non-incentivized
Perforated/bleeding ulcer	K20	Oesophagitis	Acute	
Perforated/bleeding ulcer	K21	Gastro-oesophageal reflux disease	Acute	
Perforated/bleeding ulcer	K25.0–K25.2	Gastric ulcer	Acute	Non-incentivized
Perforated/bleeding ulcer	K25.4–K25.6	Gastric ulcer	Acute	Non-incentivized
Perforated/bleeding ulcer	K26.0–K26.2	Duodenal ulcer	Acute	Non-incentivized
Perforated/bleeding ulcer	K26.4–K26.6	Duodenal ulcer	Acute	Non-incentivized
Perforated/bleeding ulcer	K27.0–K27.2	Peptic ulcer, site unspecified	Acute	Non-incentivized
Perforated/bleeding ulcer	K27.4–K27.6	Peptic ulcer, site unspecified	Acute	Non-incentivized
Perforated/bleeding ulcer	K28.0–28.2	Gastrojejunal ulcer	Acute	Non-incentivized
Perforated/bleeding ulcer	K28.4–K28.6	Gastrojejunal ulcer	Acute	Non-incentivized
Pyelonephritis and kidney/urinary tract infections	N10	Acute tubulo-interstitial nephritis	Acute	Non-incentivized
Pyelonephritis and kidney/urinary tract infections	N11	Chronic tubulo-interstitial nephritis	Acute	Non-incentivized
Pyelonephritis and kidney/urinary tract infections	N12	Tubulo-interstitial nephritis, not specified as acute or chronic	Acute	Non-incentivized
Pyelonephritis and kidney/urinary tract infections	N13.6	Pyonephrosis	Acute	Non-incentivized
Pyelonephritis and kidney/urinary tract infections	N15.9	Renal tubulo-interstitial disease, unspecified	Acute	
Pyelonephritis and kidney/urinary tract infections	N30.0	Acute cystitis	Acute	Non-incentivized
Pyelonephritis and kidney/urinary tract infections	N30.8	Other cystitis	Acute	Non-incentivized
Pyelonephritis and kidney/urinary tract infections	N30.9	Cystitis, unspecified	Acute	Non-incentivized
Pyelonephritis and kidney/urinary tract infections	N39.0	Urinary tract infection, site not specified	Acute	
Stroke	I61	Intracerebral haemorrhage		Incentivized
Stroke	I62	Other nontraumatic intracranial haemorrhage		Incentivized
Stroke	I63	Cerebral infarction		Incentivized
Stroke	I64	Stroke, not specified as haemorrhage or infarction		Incentivized
Stroke	I66	Occlusion and stenosis of cerebral arteries, not resulting in cerebral infarction		Incentivized
Stroke	I67.2	Cerebral atherosclerosis		Incentivized
Stroke	I69.8	Sequelae of other and unspecified cerebrovascular diseases		Incentivized
Stroke	R47.0	Dysphasia and aphasia		Incentivized

Note. The set of codes defining All ACSCs is the union of sets of codes defining chronic and acute ACSC [13] and incentivised and non-incentivise ACSCs [14]. Incentivised ACSCs are those whose care was incentivised under the QOF in all years 2004 to 2017.

For peer review only - http://bmjopen.bmj.com/site/about/guidelines.xhtml

Table A3: Number and annual growth rate of ACSC emergency admissions

	All ACSCs		Incentivised	
	N	Growth rate	N	Growth rate
**2004**	1955617		1111378	
**2005**	2004987	2.52%	1098023	-1.20%
**2006**	2034383	1.47%	1088389	-0.88%
**2007**	1947115	-4.29%	1012077	-7.01%
**2008**	2119358	8.85%	1086936	7.40%
**2009**	2193660	3.51%	1076740	-0.94%
**2010**	2303279	5.00%	1104581	2.59%
**2011**	2329548	1.14%	1098469	-0.55%
**2012**	2460668	5.63%	1135639	3.38%
**2013**	2490974	1.23%	1127694	-0.70%
**2014**	2427684	-2.54%	1145161	1.55%
**2015**	2523981	3.97%	1169832	2.15%
**2016**	2498565	-1.01%	1060092	-9.38%
**2017**	2508552	0.40%	1088585	2.69%
**2004 to 2017**		**28.27%**		**-2.05%**

Note. See Table A5 for a list of ICD10 codes for ACSCs. As with other studies[2,3] we found that 2007 (financial year 2007/8) was peculiar in that the number of ACSCs fell by 4.3%. This may be a result of changes in coding following the roll out of a prospective pricing regime for hospitals which linked payment to the number (and type) cases treated. There was an anomalously large fall in ACSCs classified as non-incentivised using the definitions in Harrison et al. (2014)[15] in 2014 (financial year 2014/15).

For peer review only

For peer review only - http://bmjopen.bmj.com/site/about/guidelines.xhtml

Table A4 : Yearly average local correlation of ISARs

	Global Index
2004	0.527
2005	0.500
2006	0.527
2007	0.576
2008	0.606
2009	0.576
2010	0.596
2011	0.572
2012	0.570
2013	0.536
2014	0.596
2015	0.612
2016	0.627
2017	0.446

*Note. ISAR*s: ACSC admissions indirectly standardised by age and gender. Moran's Global I is a measure of the average degree of correlation of a practice's ISAR with those of local practices. It was calculated using a 5 nearest neighbours row standardised weight matrix. The statistics are significant ($p \leq 0.0001$) in every year. Results using other spatial weight matrices are similar.

For peer review only

For peer review only - http://bmjopen.bmj.com/site/about/guidelines.xhtml

Table A5: Clustering of ISARs 2004 – 2017

Year	Spatial clusters	Practices	%	Mean	SD	min	max
2004	HH	722	8.82%	172.21	36.94	114.85	550.62
2004	LL	309	3.77%	48.14	10.97	3.95	72.13
2004	n.s.	7157	87.41%	94.83	28.47	0	367.22
2005	HH	746	9.21%	171.79	33.61	118.62	564.09
2005	LL	378	4.66%	46.14	12.82	5.59	72.84
2005	n.s.	6979	86.13%	96.73	27.23	2.53	316.07
2006	HH	768	9.52%	173.75	35.41	123.19	501.43
2006	LL	381	4.72%	45.79	11.73	7.21	71.22
2006	n.s.	6918	85.76%	95.92	27.36	0	269.55
2007	HH	750	9.37%	164.68	33.63	112.45	419.83
2007	LL	586	7.32%	43	10.6	10.75	68.45
2007	n.s.	6671	83.31%	92.24	26.41	0	234.44
2008	HH	783	9.82%	175.39	34.95	120.45	489.84
2008	LL	581	7.29%	38.99	12.5	5.19	74.18
2008	n.s.	6611	82.90%	98.33	27.73	2.72	243.53
2009	HH	756	9.53%	176.98	38.59	125.94	629.06
2009	LL	583	7.35%	39.48	13.57	7.94	73.17
2009	n.s.	6590	83.11%	100.68	27.31	12.21	289.38
2010	HH	807	10.15%	183.19	38.89	132.05	721.78
2010	LL	612	7.70%	44.25	14.03	8.26	75.74
2010	n.s.	6531	82.15%	103.67	28.22	13.44	294.17
2011	HH	768	9.76%	178	36.47	124.5	557.78
2011	LL	552	7.01%	46.91	13.33	8.59	76.13
2011	n.s.	6552	83.23%	102.93	27.77	19.46	292.91
2012	HH	762	9.71%	185.05	37.95	131.27	610.57
2012	LL	541	6.89%	48.68	13.42	9.99	79.63
2012	n.s.	6545	83.40%	107.25	28.75	25.17	325.24
2013	HH	673	8.67%	184.94	37.82	131.76	625.98
2013	LL	471	6.07%	47.6	14.47	12.49	79.89
2013	n.s.	6615	85.26%	106.93	30.62	25.23	962.19
2014	HH	712	9.40%	176.11	31.14	122.31	360.88
2014	LL	532	7.03%	38.68	13.8	8.28	75.21
2014	n.s.	6328	83.57%	101.74	29.8	12.17	881.62
2015	HH	702	9.51%	178.88	30.08	117.09	341.99
2015	LL	475	6.44%	32.8	16.02	0	71.57
2015	n.s.	6201	84.05%	103.08	29.92	14.38	887.25
2016	HH	723	9.91%	173.47	31.74	123.46	450.75
2016	LL	519	7.11%	33.03	14.94	4.18	66.13
2016	n.s.	6057	82.98%	99.25	28.53	12.21	675.46
2017	HH	576	7.85%	179.73	40.21	118.18	558.96
2017	LL	296	4.03%	31.24	13.81	1.68	60.88
2017	n.s.	6468	88.12%	97.09	34.2	0	954.53

ISARs: ACSC admissions indirectly standardised age and gender. Local clusters are identified using Moran's Local Index of Spatial Association. ns: LISA for practice is not statistically significant at 1%.

For peer review only

Table A6: Transition probabilities (%) between spatial cluster between 2004 and 2017

		Type of cluster in 2017			
		LL	n.s.	HH	Total
Type of cluster in 2004	LL	69.28	30.69	0.03	100
	n.s.	2.4	94.34	3.26	100
	HH	0.06	29.92	70.02	100

Note. n.s. local clustering not significant.

For peer review only

Supplementary Figures

Figure A1 – Practice level ISAR ACSC emergency admission 2017 for stroke, for congestive heart failure, and for flu and pneumonia

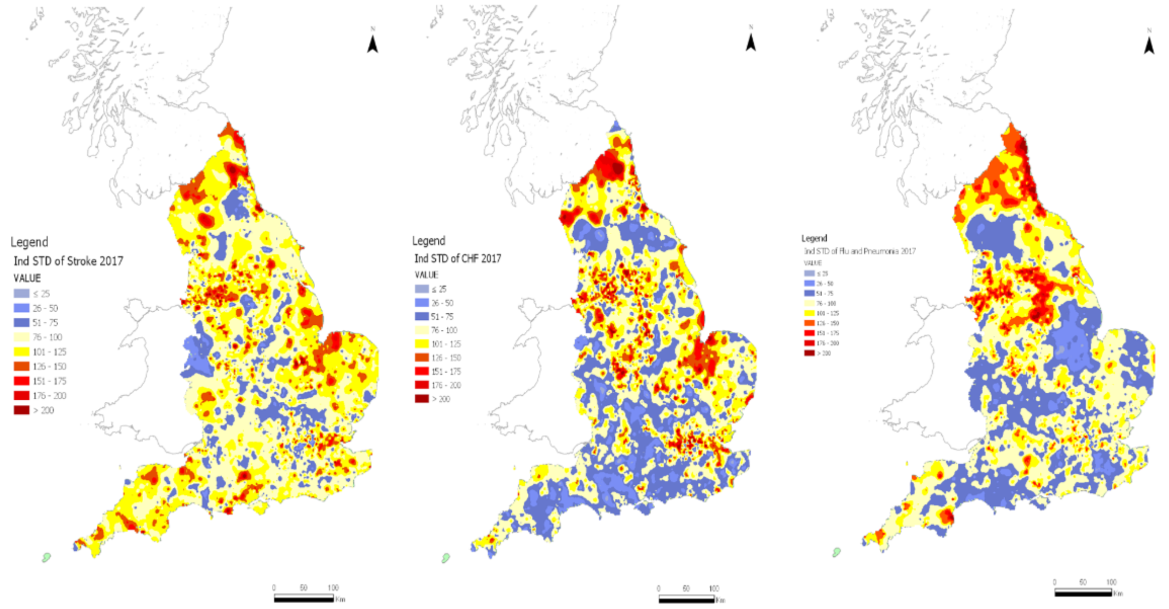

Note: ACSC rates are indirectly standardised by age and gender with expected rates for the reference population computed from 2017 data.

For peer review only - http://bmjopen.bmj.com/site/about/guidelines.xhtml

Figure A2 – Change in ACSC ISARs 2004 to 2017

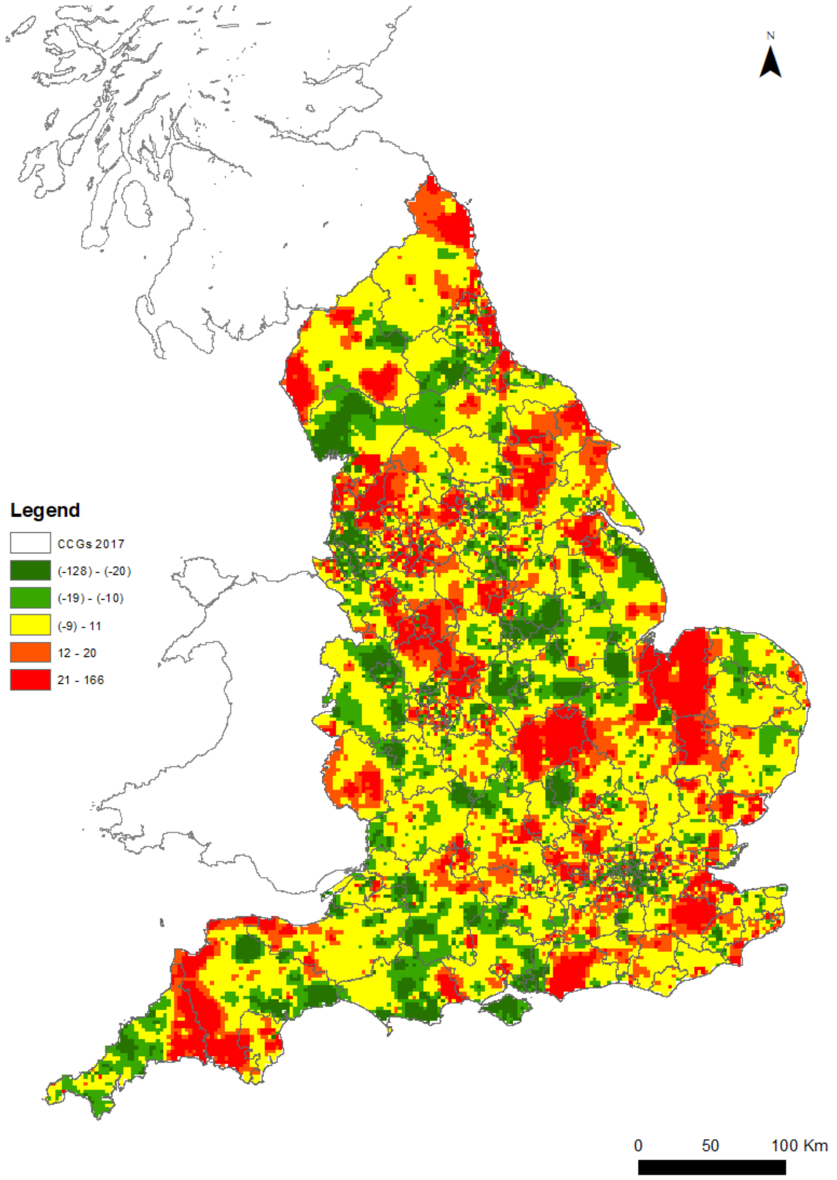

Note. ACSC emergency admissions are indirectly standardised by age and gender with expected rates for the reference population computed from data for all practices for all years 2004 to 2017.

Areas in red indicate increases in admission ratios over the observation period, while areas in green indicate decreases. Some areas with high ACSC ratios in 2004 improved over time, for example areas in and around Liverpool and Hull. Other areas with initial high admission rates did not experience a decrease, for example areas in and around Sunderland and Greater Manchester. Conversely, areas observed to have a relatively low ACSC rates in 2004, for example, Plymouth and York, observed a notable increase to 2017.

For peer review only - http://bmjopen.bmj.com/site/about/guidelines.xhtml

STROBE Statement—checklist of items that should be included in reports of observational studies

	Item No	Recommendation	Page No
**Title and abstract**	1	(*a*) Indicate the study's design with a commonly used term in the title or the abstract	1
		(*b*) Provide in the abstract an informative and balanced summary of what was done and what was found	2
**Introduction**			
Background/rationale	2	Explain the scientific background and rationale for the investigation being reported	4-5
Objectives	3	State specific objectives, including any prespecified hypotheses	4-5
**Methods**			
Study design	4	Present key elements of study design early in the paper	4
Setting	5	Describe the setting, locations, and relevant dates, including periods of recruitment, exposure, follow-up, and data collection	5-6
Participants	6	(*a*) *Cohort study*—Give the eligibility criteria, and the sources and methods of selection of participants. Describe methods of follow-up<br>*Case-control study*—Give the eligibility criteria, and the sources and methods of case ascertainment and control selection. Give the rationale for the choice of cases and controls<br>*Cross-sectional study*—Give the eligibility criteria, and the sources and methods of selection of participants	5-6
		(*b*) *Cohort study*—For matched studies, give matching criteria and number of exposed and unexposed<br>*Case-control study*—For matched studies, give matching criteria and the number of controls per case	5-6
Variables	7	Clearly define all outcomes, exposures, predictors, potential confounders, and effect modifiers. Give diagnostic criteria, if applicable	6
Data sources/ measurement	8*	For each variable of interest, give sources of data and details of methods of assessment (measurement). Describe comparability of assessment methods if there is more than one group	*6 -9*
Bias	9	Describe any efforts to address potential sources of bias	7 -9
Study size	10	Explain how the study size was arrived at	6
Quantitative variables	11	Explain how quantitative variables were handled in the analyses. If applicable, describe which groupings were chosen and why	7- 9
Statistical methods	12	(*a*) Describe all statistical methods, including those used to control for confounding	7-9
		(*b*) Describe any methods used to examine subgroups and interactions	6
		(*c*) Explain how missing data were addressed	6
		(*d*) *Cohort study*—If applicable, explain how loss to follow-up was addressed<br>*Case-control study*—If applicable, explain how matching of cases and controls was addressed<br>*Cross-sectional study*—If applicable, describe analytical methods taking account of sampling strategy	7-9
		(*e*) Describe any sensitivity analyses	

Continued on next page

For peer review only - http://bmjopen.bmj.com/site/about/guidelines.xhtml

For peer review only

**Results**			
Participants	13*	(a) Report numbers of individuals at each stage of study—eg numbers potentially eligible, examined for eligibility, confirmed eligible, included in the study, completing follow-up, and analysed	n.a.
		(b) Give reasons for non-participation at each stage	n.a.
		(c) Consider use of a flow diagram	n.a.
Descriptive data	14*	(a) Give characteristics of study participants (eg demographic, clinical, social) and information on exposures and potential confounders	n.a.
		(b) Indicate number of participants with missing data for each variable of interest	n.a.
		(c) *Cohort study*—Summarise follow-up time (eg, average and total amount)	n.a.
Outcome data	15*	*Cohort study*—Report numbers of outcome events or summary measures over time	n.a.
		Case-control study—Report numbers in each exposure category, or summary measures of exposure	n.a.
		Cross-sectional study—Report numbers of outcome events or summary measures	*9*
Main results	16	(*a*) Give unadjusted estimates and, if applicable, confounder-adjusted estimates and their precision (eg, 95% confidence interval). Make clear which confounders were adjusted for and why they were included	n.a.
		(*b*) Report category boundaries when continuous variables were categorized	n.a.
		(*c*) If relevant, consider translating estimates of relative risk into absolute risk for a meaningful time period	n.a.
Other analyses	17	Report other analyses done—eg analyses of subgroups and interactions, and sensitivity analyses	10-13
**Discussion**			
Key results	18	Summarise key results with reference to study objectives	13
Limitations	19	Discuss limitations of the study, taking into account sources of potential bias or imprecision. Discuss both direction and magnitude of any potential bias	14
Interpretation	20	Give a cautious overall interpretation of results considering objectives, limitations, multiplicity of analyses, results from similar studies, and other relevant evidence	14
Generalisability	21	Discuss the generalisability (external validity) of the study results	14
**Other information**			
Funding	22	Give the source of funding and the role of the funders for the present study and, if applicable, for the original study on which the present article is based	16

*Give information separately for cases and controls in case-control studies and, if applicable, for exposed and unexposed groups in cohort and cross-sectional studies.

Note: An Explanation and Elaboration article discusses each checklist item and gives methodological background and published examples of transparent reporting. The STROBE checklist is best used in conjunction with this article (freely available on the Web sites of PLoS Medicine at http://www.plosmedicine.org/, Annals of Internal Medicine at http://www.annals.org/, and Epidemiology at http://www.epidem.com/). Information on the STROBE Initiative is available at www.strobe-statement.org.

For peer review only - http://bmjopen.bmj.com/site/about/guidelines.xhtml
For peer review only
